# Infection of a tomato cell culture by *Phytophthora infestans*; a versatile tool to study *Phytophthora*-host interactions

**DOI:** 10.1186/s13007-017-0240-0

**Published:** 2017-10-25

**Authors:** Charikleia Schoina, Klaas Bouwmeester, Francine Govers

**Affiliations:** Laboratory of Phytopathology, Wageningen University and Research, Wageningen, The Netherlands

**Keywords:** MsK8, Disease, Infection, Defense responses, Gene expression, Reactive oxygen species (ROS), Cell death, Microscopy

## Abstract

**Background:**

The oomycete *Phytophthora infestans* causes late blight on potato and tomato. Despite extensive research, the *P.* *infestans*-host interaction is still poorly understood. To find new ways to further unravel this interaction we established a new infection system using MsK8 tomato cells. These cells grow in suspension and can be maintained as a stable cell line that is representative for tomato.

**Results:**

MsK8 cells can host several *Phytophthora* species pathogenic on tomato. Species not pathogenic on tomato could not infect. Microscopy revealed that 16 h after inoculation up to 36% of the cells were infected. The majority were penetrated by a germ tube emerging from a cyst (i.e. primary infection) while other cells were already showing secondary infections including haustoria. In incompatible interactions, MsK8 cells showed defense responses, namely reactive oxygen species production and cell death leading to a halt in pathogen spread at the single cell level. In compatible interactions, several *P.* *infestans* genes, including RXLR effector genes, were expressed and in both, compatible and incompatible interactions tomato genes involved in defense were differentially expressed.

**Conclusions:**

Our results show that *P.* *infestans* can prosper as a pathogen in MsK8 cells; it not only infects, but also makes haustoria and sporulates, and it receives signals that activate gene expression. Moreover, MsK8 cells have the ability to support pathogen growth but also to defend themselves against infection in a similar way as whole plants. An advantage of MsK8 cells compared to leaves is the more synchronized infection, as all cells have an equal chance of being infected. Moreover, analyses and sampling of infected tissue can be performed in a non-destructive manner from early time points of infection onwards and as such the MsK8 infection system offers a potential platform for large-scale omics studies and activity screenings of inhibitory compounds.

**Electronic supplementary material:**

The online version of this article (doi:10.1186/s13007-017-0240-0) contains supplementary material, which is available to authorized users.

## Background

Plants are constantly facing potential microbial attackers that can cause disease, such as bacteria, fungi and oomycetes. In order to fend off pathogens and circumvent infection, plants have developed several defense mechanisms like cell wall thickening, reactive oxygen species (ROS) production and programmed cell death. These plant immune responses are often initiated by plasma membrane-spanning surface receptors as well as cytoplasmic receptors recognizing pathogen-derived molecules. Furthermore, timely transcriptional regulation of genes involved in pathogen recognition and genes involved in plant defense play an important role [1, 2]. The pathogen-derived molecules include effectors secreted by the pathogens to promote infection [3], and microbe-associated molecular patterns (MAMPs) that are recognized by plants and trigger defense responses [2, 4]. Well-known examples of MAMPs are the bacterial flagellin, fungal chitin and oomycete elicitins such as INF1 [5]. Induced defence responses can enable the plant to circumvent infection.

Plant diseases caused by oomycetes such as downy mildews and *Phytophthora* spp. cause large losses in crop production and substantial damage in natural habitats. The genus *Phytophthora* includes over a hundred species, of which some have a limited host range while others have a much broader host range [6]. *Phytophthora* *infestans* and *Phytophthora* *sojae* are two well-studied species with a narrow host range. *P.* *infestans* causes late blight disease and only infects potato and tomato. *P.* *sojae* causes stem and root rot and has just one host, soybean. In contrast, *Phytophthora* *capsici* has a very broad host range comprising more than 200 plants, mainly *Solanaceae* and *Cucurbitaceae* spp. Similarly, *Phytophthora* *palmivora* can infect many different plants species, including oil palm, cacao, and several vegetable crops like tomato. *Phytophthora* *parasitica* is another broad host range pathogen infecting the foliage, fruits or roots of plant species in more than 250 genera [7]. A typical class of effectors secreted by *Phytophthora* spp. are the so called RXLR effectors. They all have a conserved RXLR motif that has a function in translocating these effectors into the host cells [8]. For several RXLR effectors it has been shown that they are involved in suppressing plant defense and/or promoting infection [1, 9–11].

So far, studies exploring the *P.* *infestans*-host interaction are mainly based on infections of leaves of potato and tomato, as well as *N.* *benthamiana* [12]. Leaf infections resulting from drop inoculations have the advantage that the development of the expanding lesions and the lesion growth rate can be followed over time. The disadvantage however, is that an expanded lesion is composed of different zones, ranging from a biotrophic zone at the edge of the lesion to a necrotrophic zone in the center. This zonation makes it difficult to collect material of only one specific infection stage [13]. Moreover, due to the thickness of the leaves with a multilayer of cells and the autofluorescence of chlorophyll, microscopy studies are quite challenging. In analogy with studies on mammalian systems where in vitro cell cultures are frequently used as model for the in vivo situation, one could try to eliminate the disadvantages of in planta studies by using an in vitro system. Single cells could allow a more synchronized infection process and are better accessible for microscopy.

In several studies focused on plant-microbe interactions, cell suspension cultures have been utilized instead of whole plants. Cell suspensions have also been successfully used as a model system to study signalling pathways or the effect of exogenous compounds on plant cells. A very popular stable plant cell line maintained in suspension is the tobacco cell line BY-2 [14] that has been used to study plant responses to biotic stress. For example, upon inoculation with zoospores of different *P.* *nicotianae* strains it was found that reactive oxygen species (ROS) were produced by the BY-2 cells in an incompatible interaction with a non-pathogenic strain [15, 16]. BY-2 cells were also used to study the potential of a non-pathogenic *Streptomyces* sp. as biocontrol agent against *Pectobacterium* spp. [17]. Another example is the use of Arabidopsis cell suspension cultures for transcriptomic analysis with a focus on phosphoinositide-dependent phospholipase C (PI-PLC) regulated gene expression [18]. Tomato cell suspension cultures have been used to study responses to abiotic stress factors. Exposure to low oxygen activated fermentative metabolism and sugar alcohol synthesis while inhibiting the activity of the tricarboxylic acid (TCA) cycle and the biosynthesis of metabolites such as organic acids, amino acids and sugars [19]. Aimé et al. [20] used cell suspensions from the tomato cultivar Montfavet to study expression of genes encoding Pathogenesis-Related (PR) proteins upon inoculation with a pathogenic strain of the soil-borne fungal pathogen *Fusarium oxysporum* f. sp. *lycopersici* and a biocontrol strain of *F.* *oxysporum* and found a lower PR gene expression in the presence of the biocontrol strain compared to the pathogenic strain. A similar pattern was found in intact tomato plants pointing towards priming as mode of action of the biocontrol strain. Rice cell suspensions have been used to study responses upon treatment with an elicitor from the rice blast fungus *Magnaporthe* *oryzae* and this revealed that the elicitor causes metabolic alterations in the rice cells [21].

Cell suspensions have also been used to study plant responses to *Phytophthora* spp. For example, parsley cells were used to monitor cell death and changes in cell structural components upon infection with *P.* *infestans* [22]. Moreover, potato cell suspensions established from stems and microtubers from the cultivar Bintje were used to study the responses to *P.* *infestans* culture filtrates, such as acidification of the cell culture medium and lipoxygenase induction [23]. In a recent study, the induction of defense responses to various pathogens (*P.* *infestans*, *Verticillium* *dahliae*, *Spongospora* *subterranea*, and *Colletotrichum* *coccodes*) was studied using potato and *Arabidopsis* cells suspensions. It was shown that treatments with elicitors or zoospores from *P.* *infestans*, as well as exposure to other pathogens, induce alkalinisation of medium, ROS production and induction of defense related genes [24]. In addition, defense responses, including ROS production, defense gene expression and MAPK activation were studied in the interaction between *P.* *capsici* and *Capsicum* *chinense* cell suspensions [25].

It should be noted that apart from studies with the BY-2 cell line, all of the studies described above made use of unstable cell lines with a limited life span. Unlike many human and animal cell lines, plant cells cannot easily be maintained as stable cell lines. Exceptions are the tobacco BY-2 cell line and the tomato MsK8 cell line that was utilized in this study. The MsK8 cell line (hereafter referred to as MsK8 cells) is a stable cell suspension culture that was developed as a cell line representative for tomato [26]. The majority of tomato genotypes are not amenable for generation of in vitro suspension cultures and several crosses and backcrosses were needed to find a line that had the desired characteristics. A cross between *Solanum* *lycopersicum* VF11 and K93 (F3 generation of *S.* *lycopersicum* × *S.* *peruvianum*) led to the selection of one plant in the F1 generation named MsK93. Two backcrosses with the parental line VF11 resulted in a F3 generation with one line named MsK8 that could be maintained as cell suspensions. So far, MsK8 cells have been used for monitoring defense responses upon treatment with pathogen elicitors, including chitin and the flagellin peptide flg22, and the cells have shown similar defense responses as in intact tomato plants [27–29].

The aim of this study was to develop an in vitro infection system that offers consistent, synchronized infections by *P. infestans* and to test its suitability for microscopy and histochemical studies. For this purpose, the stable tomato cell suspension culture MsK8 was tested as a host for *Phytophthora*. Tomato is a natural host of *P. infestans* and therefore MsK8 cells were preferred rather than tobacco BY-2 cells. MsK8 cells were inoculated with different *Phytophthora* spp. and strains, and the infection process was monitored over time by microscopy and histochemical staining. Detailed studies of the different *P.* *infestans* infection stages were performed in order to estimate the levels of infection and the resemblance to intact leaf infection. Furthermore, defense responses of the MsK8 cells upon inoculation were studied, such as ROS production and induction of expression of several defense-related genes.

## Results and discussion

### Tomato cells growing in suspension can host *Phytophthora infestans*

**Fig. 1:**
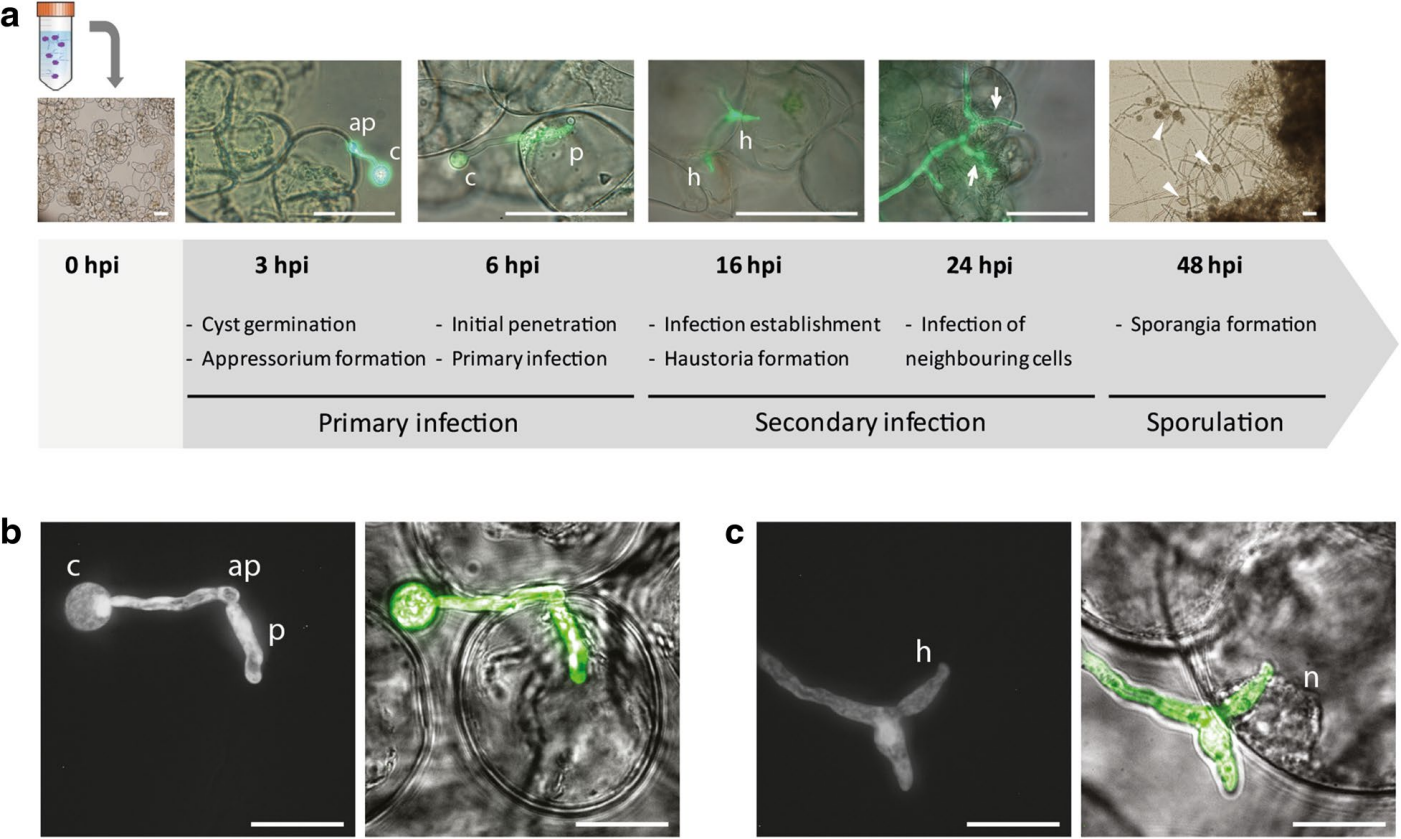
MsK8 cells are successfully infected by *Phytophthora* *infestans*. **a** Timeline of MsK8 cells infected with *P.* *infestans* strain 14-3-GFP. At time point 0 zoospores were added to MsK8 cells. Bars represent 100 μm. **b**, **c** Microscopic images (left panels: epifluorescent, right panels: bright field). **b** Primary infection and penetration of a MsK8 cell at 6 hpi. **c** Fully developed haustorium in an infected cell and relocation of the nucleus to the penetration point at 16 hpi. *ap* appressorium; *c* cyst; *h* haustorium; *hpi* hours post inoculation; *n* nucleus; *p* penetration peg. Arrows and arrowheads point to secondary infections and sporangia, respectively. Bars represent 50 μm

In order to investigate if MsK8 cells are susceptible to *P.* *infestans*, a fluorescent strain was used for inoculation and the infection process was monitored by microscopy (Additional file 1: Table S1). Zoospores from *P.* *infestans* 14-3-GFP were mixed with MsK8 cells in a 1:1 ratio. This mixture was placed on a shaking platform in the dark at RT to provide optimal conditions for both the pathogen and the host. Infection progress was monitored at different time points. Germinating cysts were found at 3 h post inoculation (hpi) while appressorium formation and initial penetrations occurred at 6 hpi (primary infections) (Fig. 1a, b). At 16 hpi, hyphae from infected cells were observed to invade neighboring cells forming secondary infections, where formation of haustoria and relocation of the nucleus to the infection point were observed (Fig. 1a, c). At 48 hpi formation of sporangiophores and sporangia was observed, suggesting successful infection of MsK8 cells and completion of the life cycle (Fig. 1a and Additional file 2: Figure S1A). In order to determine the optimal inoculum density for obtaining the maximum number of infected cells, a range of zoospore concentrations was used. A concentration of 10^5^ zoospores/mL gave the highest percentages of infected cells (Additional file 3: Table S2). In addition to *P.* *infestans* strain 14-3-GFP, two other *P.* *infestans* strains (IPO-C and T20-2) were used as inoculum (Additional file 1: Table S1). It appeared that also these two strains are capable of infecting the MsK8 cells. To compare the three strains, the infection efficiency was quantified. To this end, infected cells, i.e. cells with primary (first penetration into a single cell) and secondary infections (hypha expanding from infected to neighbouring cells), as well as cells containing haustoria were counted (Table 1). It was found that around 36% of the cells were infected by *P.* *infestans* 14-3-GFP. From the infected cells, 73% had primary infections and 26% secondary infections, from which 21% contained haustoria. Cells inoculated with strain IPO-C showed a lower percentage of infected cells (25%), but the percentages of primary and secondary infections were similar to those obtained upon inoculation with strain 14-3-GFP. MsK8 cells inoculated with strain T20-2 showed the lowest percentage of infected cells, around 16%, with a lower percentage of infected cells harbouring haustoria (Table 1). In all cases, formation of sporangia was observed at 48 hpi.

**Table 1:** Infection efficiency of various *Phytophthora* species and strains on MsK8 cells

Percentage	*P. infestans*			*P. capsici*			*P. palmivora*	*P. sojae*	*P. parasitica*
	14-3-GFP	IPO-C	T20-2	LT263	LT3239	LT51	GFP3	P6497	H1111
Infected MsK8 cells^a^	36.4 ± 1.2	25.4 ± 0.4	16.6 ± 2.3	7.0^e^ ± 4.5	0	34.0 ± 3.4	39.4 ± 1.3	0	0
Primary infection^b^	73.6 ± 3.6	73.2 ± 4.0	52.0 ± 2.5	–	–	55.3 ± 2.8	26.4 ± 2.1	–	–
Secondary infection^c^	26.4 ± 1.4	26.8 ± 2.7	48.0 ± 2.6	–	–	44.7 ± 1.1	73.6 ± 2.6	–	–
Cells containing haustoria^d^	21.4 ± 1.1	11.8 ± 1.8	7.1 ± 0.9	–	–	7.0 ± 0.6	7.1 ± 1.5	–	–

The efficiency was quantified by determining the percentage of infected cells at 16 h post inoculation. For each sample a total of 500 cells was monitored in triplicate

^a^Cells that had been penetrated by *Phytophthora* were counted as infected

^b^Infected cells due to primary infection i.e. penetrated by germ tubes emerging from cysts

^c^Infected cells due to secondary infection i.e. penetrated by hyphae expanding from a neighbouring infected cell

^d^Infected cells containing haustoria

^e^Attachment of hyphae and scarce initial penetrations with no further growth of hyphae in the cell

In infected potato and tomato leaves infection is usually established at 48 hpi and sporulation is first observed after 3–4 days post inoculation [13, 30]. In the MsK8 cells, however, the infection progress was clearly faster (Fig. 1a) suggesting that MsK8 cells can be more easily penetrated by *P.* *infestans*, likely due to the lack of a cuticle and a differentiated epidermal cell layer. In conclusion, *P.* *infestans* is able to consistently successfully infect the MsK8 cells and complete its life cycle in these host cells. This justifies further analyses of the MsK8 cell suspension line for its potential as alternative infection system for *P.* *infestans*.

### Tomato cells growing in suspension can host other *Phytophthora* species but not all

To determine whether MsK8 cells can be infected by other *Phytophthora* spp., MsK8 cells were inoculated with strains from four other *Phytophthora* species namely *P.* *capsici, P.* *palmivora*, *P.* *sojae* and *P.* *parasitica* (Additional file 1: Table S1). Inoculations with *P.* *capsici* LT263 showed that germinating cysts are capable to attach and penetrate the cell. Eventually however, infection did not proceed, despite the fact that tomato is a host for this strain [31]. Based on microscopic observations LT263 attempted to infect the cells, suggesting that this strain is less aggressive on MsK8 cells or that it cannot circumvent the induced defense mechanisms of MsK8 cells. To further investigate this, two other *P.* *capsici* strains were used that can also infect tomato plants. Strain LT3239 was not capable of infecting MsK8 cells, but strain LT51 was. The latter was able to establish infection, form haustoria and develop sporangia while the percentages of cells showing primary or secondary infection or haustoria were similar to those obtained after *P.* *infestans* inoculation (Table 1, Additional file 2: Figure S1B). Also, inoculation with *P.* *palmivora* strain GFP3 resulted in successful infection and with similar percentages of infected cells. *P.* *palmivora* is not listed as a typical tomato pathogen but has been reported to infect tomato fruits [32]. Apparently the MsK8 cells are easy victims for *P.* *palmivora*; the infection with *P.* *palmivora* was progressing faster than the infection with *P.* *infestans* with the first penetrations already observed at 3 hpi as opposed to 6 hpi with *P. infestans*. Formation of haustoria (7%) was observed at 12 hpi while at 16 hpi most cells had secondary infections (73%). Similar to *P.* *infestans*, *P.* *palmivora* could complete its life cycle in the MsK8 cells, producing sporangia after 36 hpi (Additional file 2: Figure S1C). Unlike *P.* *palmivora,* the *P.* *sojae* and *P.* *parasitica* strains tested here were not able to infect the MsK8 cells (Table 1). For *P. sojae* this is not surprising because this species has a very narrow host range. *P. parasitica* is known to cause tomato root rot [33] but this might be strain dependent. Zoospores from *P.* *sojae* strain P6497 and *P.* *parasitica* strain H1111 did encyst and germinate but there was no attachment to the MsK8 cells.

These results show that the in vitro infection system is suitable for studying interactions with other *Phytophthora* spp. besides *P.* *infestans* and moreover, that the MsK8 cells have retained their capacity to distinguish between different *Phytophthora* spp. and even between strains thus behaving similar to intact tomato plants with respect to host specificity.

### Defense responses in MsK8 cells can differentiate a compatible from an incompatible interaction

**Fig. 2:**
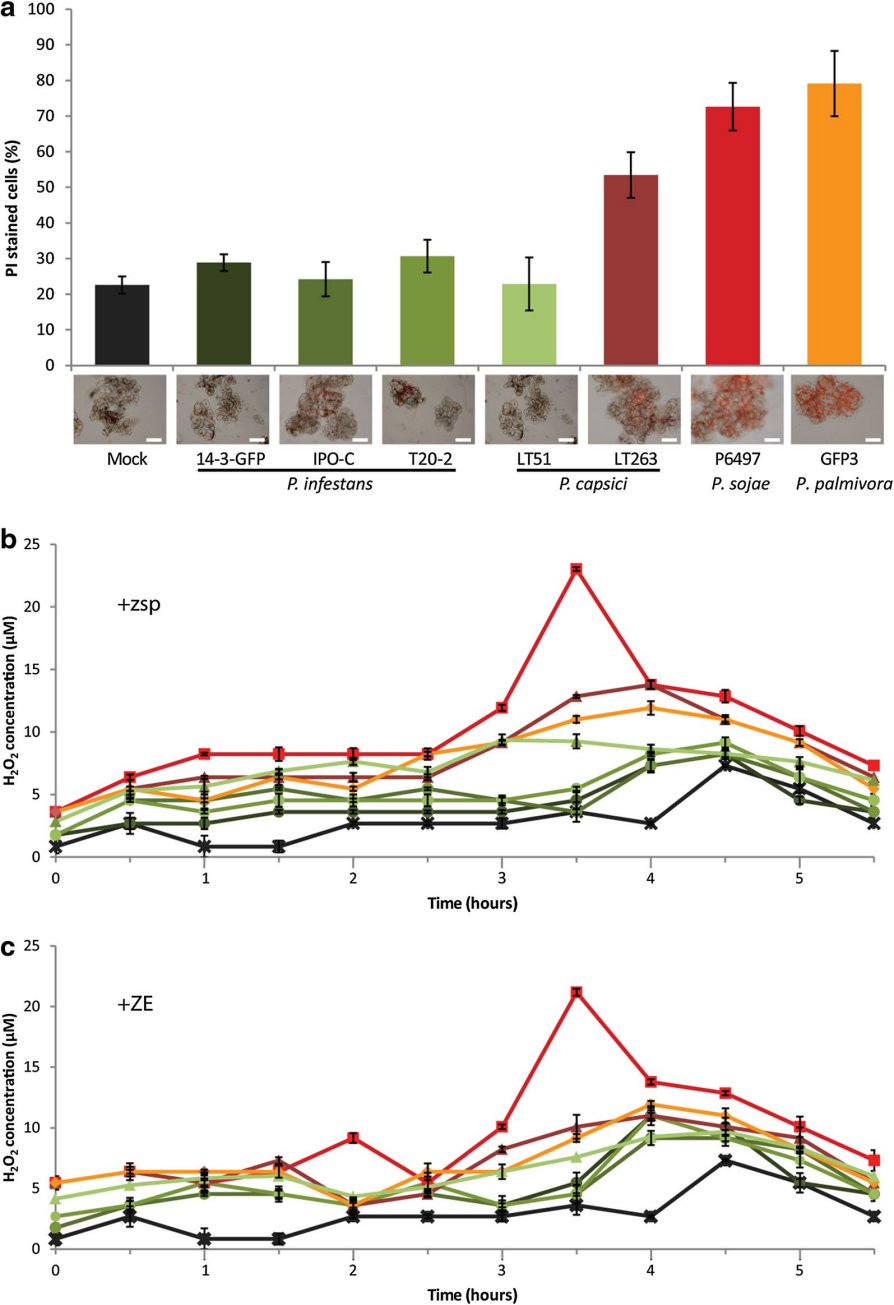
Responses of MsK8 cells to inoculation with different *Phytophthora* spp. and strains. **a** Percentage of MsK8 cells in suspension showing cell death at 16 hpi. Cell death was quantified by counting the cells stained with propidium iodine (PI), as shown in the microscopy images, and the total number of cells (set at 100%). Bars represent 200 μm. **b**, **c** ROS production by MsK8 cells measured at different time points (X-axis) after **b** inoculation with *Phytophthora* zoospores or **c** after treatment with zoospore exudate (ZE). ROS production was quantified by measuring the H_2_O_2_ concentration with the xylenol orange assay. Colors of the bars (**a**) and lines (**b**, **c**) correspond to a specific strain and species as indicated in **a**. Error bars represent standard deviation (n = 3)

To investigate how the MsK8 cells respond to exposure to *Phytophthora*, defense responses were examined. ROS accumulation and cell death are two defense mechanisms employed by the plant during the interaction with a potential pathogen [34]. First, the viability of the MsK8 cells at 20 hpi was analyzed by propidium iodine (PI) staining. It was found that the three *P.* *infestans* strains and the *P.* *capsici* strain LT51 that are compatible with the MsK8 cells, do not induce cell death. In contrast, *P.* *sojae* P6497 and *P.* *capsici* LT263, two strains that are not able to infect, did induce cell death (Fig. 2a). At 20 hpi with *P.* *sojae*, 72% of the cells were dead, and this is in line with the microscopic observations that clearly revealed an incompatible interaction (Fig. 2a). However, also in the compatible interaction of MsK8 cells with *P.* *palmivora*, a high percentage of cell death was observed, namely in 79% of the cells, and this was also visible by microscopy.

When measuring electrolyte leakage, which is an indirect method to quantify cell death, MsK8 cells inoculated with *P.* *sojae* at 20 and 24 hpi showed higher conductivity when compared to the mock treated cells indicating cell death. An increase in electrolyte leakage was also observed upon inoculation with *P.* *palmivora* from 20 hpi onwards, but to a lesser extent. However, MsK8 cells inoculated with the other *Phytophthora* spp. did not show a significant difference in conductivity compared to the mock (Additional file 4: Figure S2A), despite the fact that some of the interactions were clearly incompatible when analysed by microscopy and showed (partial) cell death.

ROS production is another marker for activation of defense responses and can be quantified using the xylenol orange assay. Measurements at different time points post inoculation revealed that inoculation of MsK8 cells with *P.* *sojae* leads to a peak in ROS production at 3.5 hpi. Similarly, Able et al. [15] observed cell death and ROS production in incompatible interactions between BY-2 cells and avirulent strains of *P.* *parasitica* var. *nicotianae*. Also in MsK8 cells inoculated with *P.* *palmivora* GFP3 and *P.* *capsici* LT263 we observed accumulation of ROS but at lower levels (Fig. 2b). Although the interaction with *P.* *palmivora* was compatible, the higher percentage of dead cells and the higher levels of ROS production compared to *P.* *infestans*, could be due to the fact that the infection process is faster and *P.* *palmivora* enters the necrotrophic stage earlier. At the time of measurement, the *P.* *palmivora*-MsK8 interaction probably had already entered the necrotrophic stage, as noticed by the microscopic observations and the cell death measurements.

**Fig. 3:**
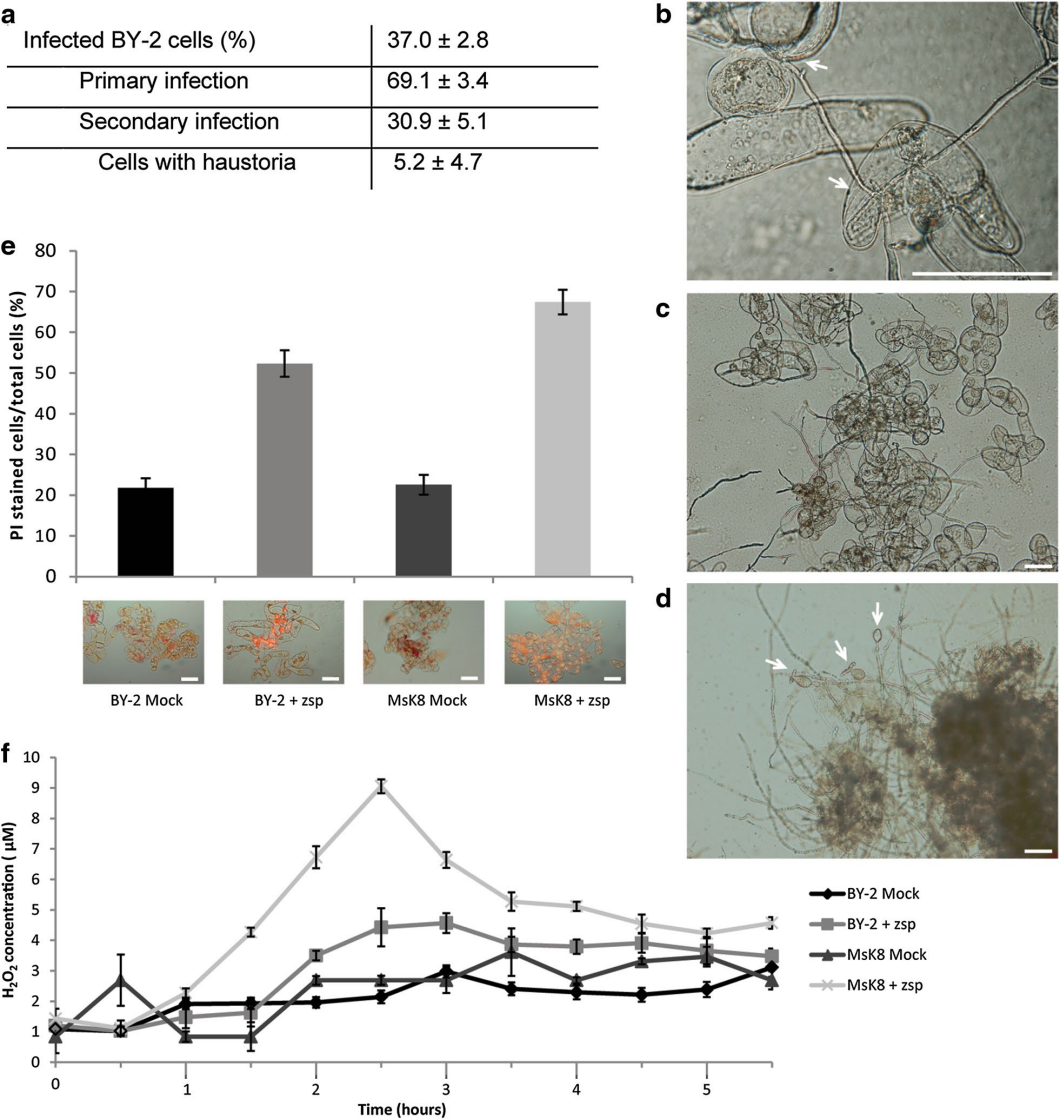
*Phytophthora* *parasitica* H1111 is able to infect BY-2 cells. **a** Quantification of *P.* *parasitica* H1111 infections on BY-2 cells at 16 hpi (n = 3, 500 cells/sample). Cells that have been penetrated by *Phytophthora* hyphae were counted as infected. **b**–**d** Microscopic images of BY-2 cells penetrated by *P.* *parasitica* hyphae at 6 hpi (**b**), secondary infection at 16 hpi (**c**) and formation of sporangia at 48 hpi (**d**). Arrows point to the sites of penetration (**b**) or sporangia (**d**). Bars represent 100 μm. **e** Percentages of BY-2 and MsK8 cells in the cell suspension showing cell death at 16 hpi. Cell death was quantified by counting the cells stained with PI (as shown in the microscopic images) and the total number of cells (set at 100%). Bars in images represent 200 μm. **f** ROS production by MsK8 and BY-2 cells measured at different time points (X-axis) after inoculation with *P.* *parasitica* zoospores. ROS production was quantified by measuring the H_2_O_2_ concentration with the xylenol orange assay. Error bars represent standard deviation (n = 3)

The fact that *P.* *parasitica,* a species that is able to infect tomato plants, did not cause successful infection of MsK8 cells, urged us to evaluate the fitness and virulence of the strain used in this study. *P.* *parasitica* also infects tobacco [35, 36] and therefore we used *P. parasitica* strain H1111 to inoculate the tobacco cell suspension BY-2. While, H1111 was unable to cause infection of MsK8 cells, it was able to infect BY-2 cells; 37% of the cells became infected and haustoria were formed in 5% of the infected cells (Fig. 3a). These percentages are similar to those obtained in the *P.* *infestans*—MsK8 interaction (Table 1). Furthermore, *P.* *parasitica* was able to complete its life cycle and produce sporangia from 48 hpi onwards (Fig. 3b–d). Cell death measurements showed an increase of dead cells upon inoculation with *P.* *parasitica*. This increase was higher for MsK8 compared to BY-2 cells at 16 hpi, depicting a difference between successful and unsuccessful infection (Fig. 3e). Measurements of ROS production showed higher amounts of H_2_O_2_ in inoculated MsK8 cells at 3.5 hpi compared to inoculated BY-2 cells, indicating a stronger defense response in MsK8 cells when challenged with the pathogen (Fig. 3f).

In summary, based on cell death and ROS production the defense responses of MsK8 cells are indicative of a compatible or incompatible interaction, depending on the *Phytophthora* species. The high levels of ROS production and cell death in the incompatible interaction with *P.* *sojae,* indicate recognition of the pathogen. On the other hand, in the compatible interaction with *P.* *infestans* the ROS production was much lower and this is in agreement with the hemi-biotrophic lifestyle of the pathogen.

### Responsiveness of MsK8 cells to *Phytophthora* spp. zoospore exudates

In order to examine if *Phytophthora* zoospores release molecules that induce defense responses in MsK8 cells, the cells were mixed with zoospore exudates (ZE) and responses were measured by quantifying ROS production and electrolyte leakage. As upon zoospore treatment, ROS production by MsK8 cells was the highest at approximately 3.5 h after mixing with ZE of *P.* *sojae* P6497 and reached similar levels (Fig. 3c). Electrolyte leakage measurements did not show an increase in conductivity for any of the samples of MsK8 cells treated with ZEs when compared to mock treated cells, not even with ZE of *P. sojae* strain P6497 of which the zoospores, when mixed with MsK8 cells, caused an increase in conductivity (Additional file 4: Figure S2B). Overall, treatments of MsK8 cells with ZE of *Phytophthora* spp. showed levels of cell death and ROS accumulation similar to zoospore treatments, suggesting that the presence of the pathogen is not prerequisite for induction of defense responses. In other studies, treatments with MAMPs such as chitin, induced defense responses in Msk8 cells [37]. Furthermore, culture filtrates from *P.* *infestans* and a species of the bacterium *Streptomyces* induced changes in pH in potato cells and ROS production in BY-2 cells, respectively [17, 23].

The most abundant protein secreted by *P. infestans* is the elicitin INF1, a small protein of 98 amino acids classified as a MAMP that elicits cell death in several *Nicotiana* spp. [12]. INF2B is larger but shares the canonical elicitin domain with INF1 [38]. In the wild potato species *S. microdontum*, elicitin recognition is mediated by the receptor-like protein ELR [39]. To study the response of MsK8 cells to elicitins the cells were exposed to the full length INF1 and the elicitin domain of INF2B proteins. The responsiveness was measured by monitoring medium alkalinization. No pH shift was observed upon treatment with INF1 or INF2B (Additional file 5: Figure S3A). On the other hand, tobacco BY-2 cells showed a response to both INF1 and INF2B (Additional file 5: Figure S3B) demonstrating that the protein preparations used do have elicitor activity. To verify the responsiveness of MsK8 cells to other MAMPs, they were treated with the flagellin peptide flg22 and this resulted in a pH shift (Additional file 5: Figure S3C). These findings confirmed previous studies by Felix et al. [28] who also observed medium alkalinization in MsK8 cells upon exposure to flg22. In conclusion, the MsK8 cells do respond to flg22 but not to elicitins and this resembles the response of intact tomato plants to these MAMPs [12, 28].

### Activation of *Phytophthora* genes during interaction with MsK8 cells

In order to investigate how *Phytophthora* responds upon encountering MsK8 cells as potential host we analysed the expression of several *Phytophthora* genes in time up to 36 hpi. These included a number of RXLR effector genes as well as genes that are proposed as marker genes for subsequent infection stages, namely *HMP1*, *NPP1* and *CDC14* (Jupe et al. [40]; Additional file 6: Table S3).

**Fig. 4:**
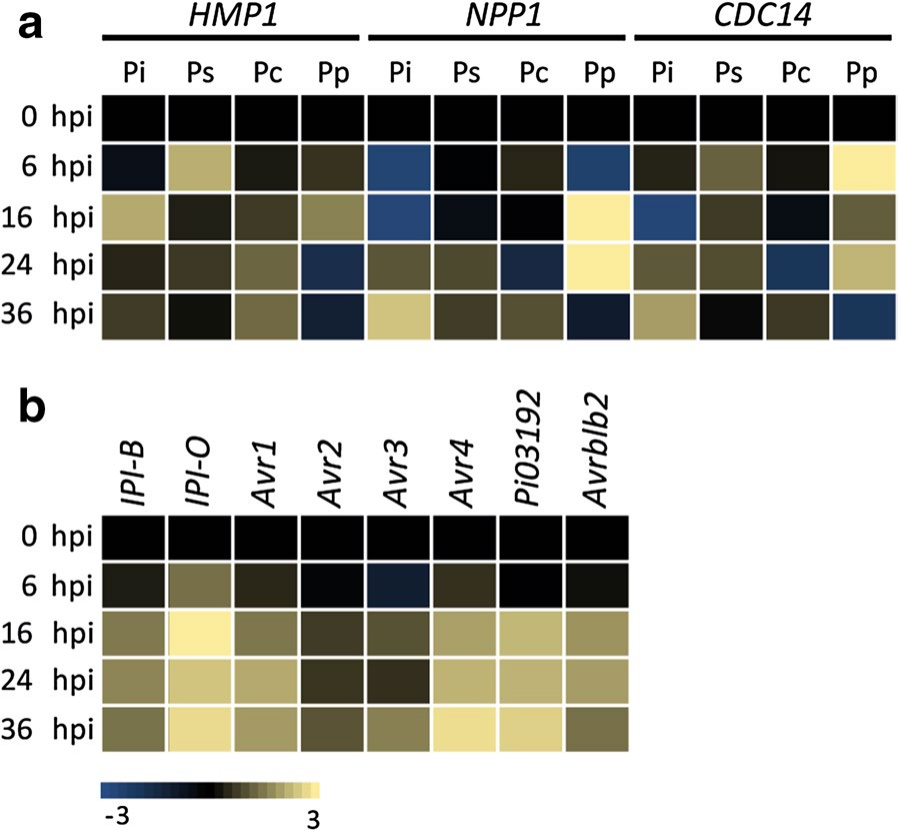
Expression profiling of *Phytophthora* genes during infection. **a** Expression of stage-specific genes *HMP1*, *NPP1* and *CDC14* upon inoculation of MsK8 cells with zoospores of *P.* *infestans* 14-3-GFP (Pi), *P.* *capsici* LT263 (Pc), *P.* *palmivora* GFP3 (Pp) and *P.* *sojae* P6497 (Ps). **b** Expression of *IPI*-*B* and various *P.* *infestans* RXLR effector genes upon inoculation of MsK8 cells with zoospores of *P.* *infestans* 14-3-GFP. Expression levels were determined by qRT-PCR and the values at each time point were calculated relative to the expression level at time point 0 (0 hpi). Expression of the actin gene *ActA* was used as endogenous control

The qRT-PCR analyses showed that in all three *P.* *infestans* strains (14-3-GFP, IPO-C and T20-2) the haustorium-specific gene *HMP1* [41] reaches the highest expression level at 16 hpi after which expression continues but decreases. *P.* *palmivora* GFP3 only shows *HMP1* expression at 16 hpi (Fig. 4a, Additional file 7: Figure S4). This is in accordance with the microscopic observations showing the formation of haustoria at that time point. However, also in *P.* *capsici* LT263 and *P.* *sojae* P6497 that are both incompatible with MsK8 cells, *HMP1* is expressed and in *P.* *sojae* P6497 the expression peaks even earlier than in *P.* *infestans* and *P.* *palmivora,* i.e. at 6 hpi, the earliest time point measured (Fig. 4). In these incompatible interactions, there are no haustoria formed indicating that *HMP1* expression does not necessarily correlate with haustoria formation. In fact, Avrora et al. [41] who first identified the gene in *P. infestans,* showed that *HMP1* is also highly expressed in germinating cysts and appressoria. This likely explains why we see *HMP1* expression in these incompatible interactions where, as described above, the cysts do germinate and make attempts to penetrate the MsK8 cells. Overall, the *HPM1* expression profiles that we observe in compatible interactions with MsK8 cells are in line with the profiles observed by others in *P.* *infestans* and *P.* *capsici* infected leaves with upregulation early during infection concurrently with haustoria formation and downregulation afterwards [41, 42]. We are not aware of studies in which *HPM1* expression has been monitored in incompatible interactions or non-host interactions.

The *NPP1* gene that encodes a necrosis-inducing protein, is upregulated at later time points than *HPM1.* Expression in the three tested *P.* *infestans* strains and in *P.* *palmivora* GFP3 that are compatible with MsK8 cells, is higher than in *P.* *capsici* LT263 and *P.* *sojae* P6497 that do not infect MsK8 cells (Fig. 4a, Additional file 7: Figure S4). In *P.* *palmivora* GFP3 expression is already high at 16 hpi, possibly due to the faster infection progress and in accordance with the cell death that is more pronounced in the *P.* *palmivora*-MsK8 interaction (Fig. 4a, Additional file 7: Figure S4). In infected tomato leaves a comparable *NPP1* expression pattern was observed with no or very low expression in early stages and upregulation in later stages [30, 40, 42, 43].

Similar to *NPP1, CDC14*, a gene encoding a cell cycle regulator, is expressed at earlier time points (6 hpi) in *P.* *palmivora* than in *P.* *infestans* where expression is first observed at 24 hpi (Fig. 4a). At 6 hpi the cysts are germinating and at 24 and 36 hpi there is mycelial growth and formation of sporangia. Ah Fong and Judelson [44] who first identified *CDC14* as a sporulation-specific gene, reported low expression in vegetative hyphae and high expression in sporangia, zoospores and cysts. As such, this explains the expression patterns that we observe here in infected MsK8 cells and that others have observed in tomato leaves infected with either with *P.* *infestans* [42] or *P.* *capsici* [40]. Also the very low expression of *CDC14* in the incompatible interaction of MsK8 with *P.* *capsici* or *P.* *sojae* is not suprising since these species do not proceed to the stage where sporulation occurs.

RXLR effectors are virulence proteins produced by *Phytophthora* species and as such, important markers for monitoring the suitability of a novel infection system. They do share the RXLR motif but apart from that, they are very diverse in sequence with every species having hundreds of different RXLR effectors each with a specific role in the infection process [45]. Moreover, RXLR effectors are highly variable among isolates, not only with respect to sequence or copy number polymorphism but also expression levels and expression dynamics. Since RXLR effectors are species-specific we limited this analysis to the three *P.* *infestans* strains included in this study. We selected seven *P.* *infestans* RXLR effector genes that have been analyzed in previous studies. In infected leaves expression usually occurs early during infection [46]. In line with that, we observed expression at variable levels of all seven RXLR effector genes at 16 hpi in *P.* *infestans* 14-3-GFP and even earlier, at 6 hpi, in *P.* *infestans* IPO-C (Fig. 4, Additional file 7: Figure S4). In the less aggressive T20-2 strain expression of the tested RXLR effector genes is overall lower and peaks later during the interaction with MsK8 cells (24 hpi) (Additional file 7: Figure S4). Differences among the three strains in timing of expression and expression level of an individual RXLR effector gene can likely be explained by the dynamics of the gene itself, in addition to the dynamics of the whole RXLR effectome. *Avr2* for example, is regulated at various levels [47]. Analyses of a large set of field isolates revealed presence/absence of the *Avr2* gene, the existence of *Avr2*-like genes, differential expression and sequence polymorphism. Wang et al. [48] showed that the *P.* *sojae* effectome is a complex network in which the interplay between effectors and the redundancy among effectors determines expression and activity of each individual RXLR effector.

Taken together our results show that in the MsK8 interactions the expression patterns of *Phytophthora* genes characteristic for early (*HMP1*) and later (*NPP1*) infection stages and for growth of the pathogen (*CDC14*), are in accordance with the microscopic observations. Moreover, induction of expression of RXLR effector genes early in the interaction shows that *P.* *infestans* recognises MsK8 cells as a suitable host.

### Expression of defense-related genes in MsK8 cells

**Fig. 5:**
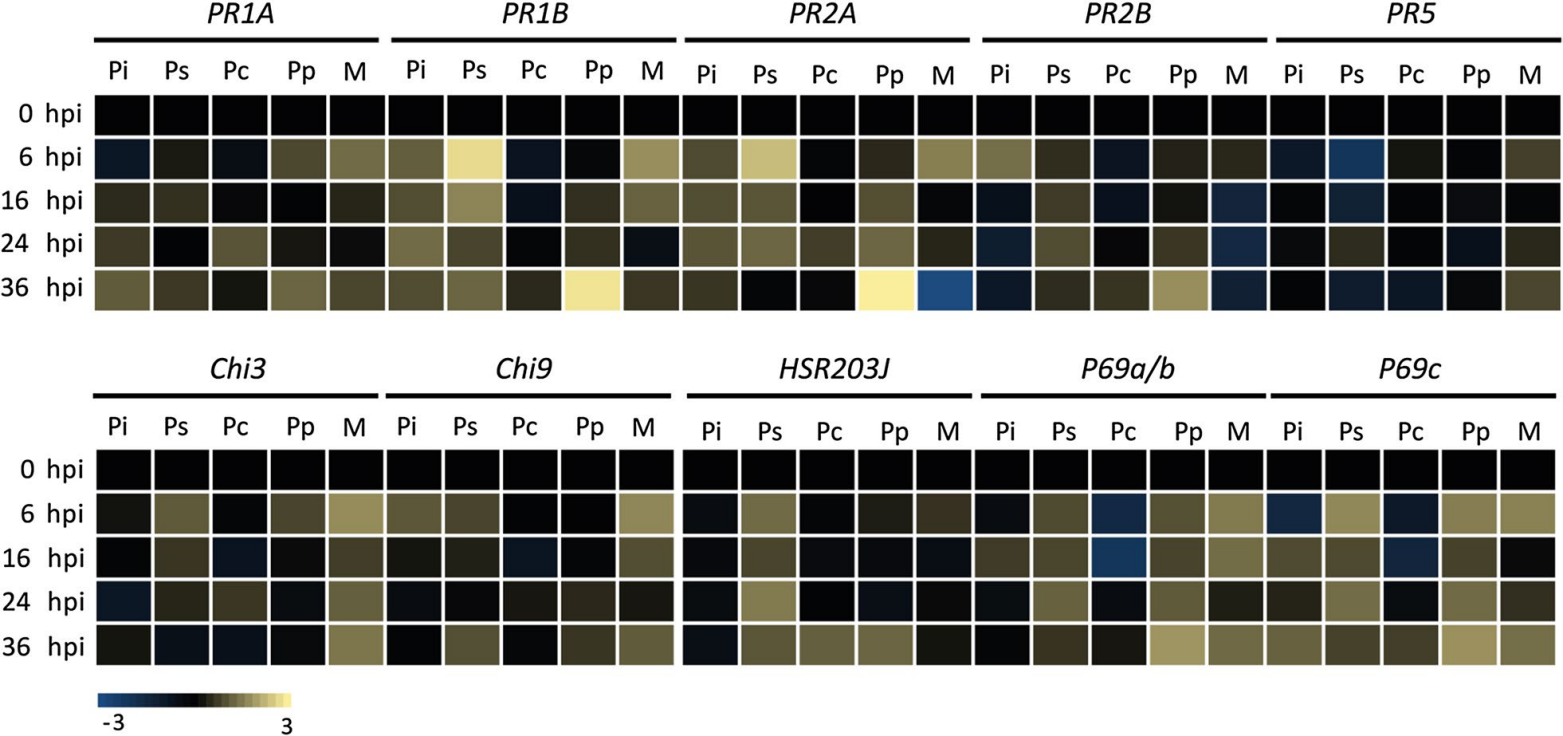
Expression profiling of tomato defense marker genes upon inoculation of MsK8 cells with zoospores of *P.* *infestans* 14-3-GFP (Pi), *P.* *sojae* P6497 (Ps), *P.* *capsici* LT263 (Pc) and *P.* *palmivora* GFP3 (Pp). Defense genes include genes encoding different pathogenesis-related proteins (PR), chitinases (Chi), a hypersensitivity marker (HSR203J) and isoforms of the subtilase P69 (P69a/b and P69c). Expression levels were determined by qRT-PCR and the values were calculated relative to the expression level at time point 0 (0 hpi). Expression of the tomato *ActA* was used as endogenous control

In order to monitor how MsK8 cells respond to inoculation with *Phytophthora*, the expression of several defense-related genes was analysed in time up to 36 hpi. This included genes encoding pathogenesis-related (PR) proteins (*PR1A*, *PR1B*, *PR2A*, *PR2B*, and *PR5*), chitinases (*Chi3* and *Chi9*), a hypersensitive response marker (*HSR203J*) and isoforms of a pathogenesis-related subtilase (*P69a/b* and *P69c*) (Additional file 6: Table S3). In mock-treated MsK8 cells that were included as control, nearly all tested defense-related genes showed an increase in expression at 6 hpi and in particular the chitinase and subtilase genes continued to be expressed at higher levels up to 36 hpi. Compared to the mock-treated controls MsK8 cells challenged with *P.* *infestans* 14-3-GFP showed a lower expression of *PR1A, PR5*, *Chi3*, *Chi9*, *HSR203J* and *P69a/b,* at all time points while *PR1B, PR2A* and *PR2B*, expression was the same or slightly upregulated. The expression of *P69c* at 6 hpi was lower than in the mock control but at 16 hpi it was slightly higher (Fig. 5). In MsK8 cells inoculated with zoospores of the other *P.* *infestans* strains (IPO-C and T20-2), a similar trend was observed (Additional file 8: Figure S5). In MsK8 cells inoculated with *P.* *palmivora* GFP3, the expression of the *PR* genes and chitinase genes was overall comparable to expression in *P.* *infestans* infected cells with the exception of a strong upregulation of *PR1A*, *PR2A* and *PR2B* at 36 hpi. Also *P69a/b* and *P69c* showed a higher expression in *P.* *palmivora* infected cells compared to *P.* *infestans* infected cells and this was already evident at the earliest time point that was monitored, i.e. 6 hpi (Fig. 5). In MsK8 cells inoculated with *P.* *capsici* LT263 hardly any changes in expression levels were observed. This is in contrast to MsK8 cells inoculated with *P.* *sojae* P6497 in which seven out of the nine defense genes analysed here showed a strong upregulation shortly after inoculation, with a peak at 6 hpi. This concerned *PR1B*, *PR2A*, *Chi3, Chi9, HSR203J*, *P69a/b* and *P69c* (Fig. 5).

To investigate if expression of defense genes is induced by molecules secreted by the pathogen, MsK8 cells were treated with ZE and expression analyses were performed in a similar manner. When comparing the expression patterns between zoospore inoculated MsK8 cells and ZE treated MsK8 cells some differences were observed. Most remarkable was the rather strong and rapid response (within 6 h) of *PR1A*, *P69a*/b and *P69c* to ZE derived from *P.* *sojae, P.* *capsici* and *P.* *palmivora* but not *P.* *infestans* (Fig. 5, Additional file 9: Figure S6). Another remarkable difference was the increased expression of these three defense related genes as well as *PR1B*, *Chi9* and *HSR203J* upon treatment with ZE from *P.* *capsici* while inoculation with *P.* *capsici* zoospores did not increase the expression of these same genes. Furthermore, it was noted that ZE of *P.* *infestans* 14-3-GFP seems to lack molecules that activate the defense genes tested here while treatment with ZE of the other two *P. infestans* strains resulted in activation of *PR1B*, *PR2A*, *PR2B* and *Chi3* (Additional file 9: Figure S6). This suggests variability among strains or, potentially, among ZE from different strains. All ZE samples were collected by the same procedure. Yet, we have no means to check the composition of the exudates in a straightforward manner. In the ROS production assays and electrolyte leakage measurements the MsK8 cells responded in a similar manner to ZE from all three *P. infestans* strains (Fig. 2, Additional file 4: Figure S2A) but with respect to defense gene expression we observe some minor differences.

Overall, our results show that there is differential expression of several defense related genes in MsK8 cells when the cells are challenged with different *Phytophthora* spp. or their ZEs. However, there is no clear correlation between the patterns that we observe and the type of interaction with the MsK8 cells, i.e. compatible versus incompatible. We could speculate that the rapid increase in defense gene expression in MsK8 cells leads to an incompatible interaction as observed upon inoculation with *P.* *sojae,* a species that is unable to circumvent the defense. However, this does not explain why the interaction between *P.* *capsici* and Msk8 cells is incompatible because there is no obvious increase in defense gene expression. In literature there are many reports showing expression profiles of defense-related genes during host-pathogen interactions. In cases where compatible and incompatible interactions are compared the overall tendency is a stronger expression in incompatible interactions. It should be noted however, that those studies often concern interactions of one host species with either a virulent or avirulent strain of the same *Phytophthora* spp. and not non-host interactions.

## Conclusion

Model pathosystems have been instrumental in extending our knowledge on how *Phytophthora* pathogens infect plants and cause disease. Leaf infection assays are routinely used to dissect the complexity of *Phytophthora*-host interactions. A disadvantage of leaf infections, however, is the occurrence of different infection stages simultaneously. In the in vitro infection system established in this study all MsK8 cells growing in suspension have an equal chance of being infected. Defined quantities of *Phytophthora* zoospores can be mixed with a standardized amount of MsK8 cells, and infection can be followed over time by microscopy. Shortly after inoculation initial penetration and primary infection of MsK8 cells were observed and this was followed by a secondary stage including haustorium formation and infection of neighbouring cells. The induced expression of infection-related genes in *P.* *infestans*, including RXLR effector genes, showed that *P.* *infestans* recognises MsK8 cells as a suitable host. During incompatible interactions, MsK8 cells mounted early defense responses, including cell death and ROS accumulation. So apart from the ability to host pathogen growth, MsK8 cells retained their capacity to perceive and counterattack *Phytophthora* pathogens in a similar way as observed in whole plants. MsK8 cells can easily be maintained under controlled conditions in a way that the status of the host tissue is comparable between experiments. The variability among infection assays was found to be minimal. This in vitro system accommodates synchronized infection allowing analyses and sampling in a non-destructive manner from early time points of infection onwards and offers a potential platform for large-scale-omics studies and activity screenings of inhibitory compounds.

## Methods

### Plant cell suspensions culture

MsK8 [26] and BY-2 cell suspensions [14] were cultured in Murashige and Skoog (MS) medium including vitamins (Duchefa Biochemie), supplemented with 30 g/L sucrose, 1 mg/L 2,4-dichlorophenoxyacetic acid (2,4-D) and 0.1 mg/L kinetin, which was set to pH 5.7 (MS-30). Cells were routinely grown in 60 mL MS-30 medium in 300 mL Erlenmeyer flasks, placed at 25 °C in the dark, on a platform shaking at approximately 100 rpm. To maintain the vigor of the cell suspensions, sub-culturing was performed every 7 days by transferring 10 mL cell suspension to flasks containing 50 mL MS-30 medium.

### *Phytophthora* spp. culturing and inoculum preparation

The *Phytophthora* strains used in this study are listed in Additional file 1: Table S1. *P.* *infestans* strains were grown on rye sucrose agar medium (RSA) at 18 °C. *P.* *capsici*, *P.* *sojae, P.* *palmivora* and *P.* *parasitica* were grown on solid V8 medium at 25 °C. *P.* *infestans* zoospores were isolated from 10 days-old sporulating mycelium which was flooded with 15 mL ice-cold water and thereafter kept at 4 °C for 3 h to induce zoospore release. Zoospores from *P.* *capsici, P.* *palmivora* and *P.* *parasitica* were obtained in a similar way. In particular, 8 days-old mycelium was flooded with 20 mL cold water and kept at room temperature (RT) for 2 h. Zoospore suspensions were filtered through a sterile 50 μm mesh. Zoospore concentrations were determined using a haemocytometer and adjusted to 10^5^ zoospores/mL. To obtain *Phytophthora* zoospore exudates (ZE), zoospore suspensions were pelleted by centrifugation (4.600×*g* for 10 min). The supernatant was collected and filtered through a 0.45 μm filter.

### Inoculation assays

Inoculations of MsK8 or BY-2 cells with *Phytophthora* zoospore suspensions were performed by mixing a sample of a 6 days-old cell culture (approx. 10^6^ cells/mL) with zoospore suspensions of 10^5^ zoospores/mL, in a 1:1 ratio. Inoculated cells were incubated at RT in the dark, shaking at 80–100 rpm. Mock and ZE treatments were performed in a similar way.

### Histochemical staining and microscopy

Cell death in treated BY-2 and MsK8 cells, was determined by staining with propidium iodine (PI) (0.05 mg/mL). The value for cell viability was inferred by the number of PI stained cells relative to the total number of cells expressed in percentages. Each measurement consisted of three technical replicates per sample and a total number of 500 cells per replicate. Microscopic observations were performed on a Nikon eclipse 90i epifluorescence microscope equipped with a Nikon DS-U2 digital imaging camera. Fluorescence was captured using a GFP-LP filter (EX 460-500, DM 505). Confocal fluorescence microscopy was performed on a Roper Spinning Disc confocal microscope (Nikon Ti microscope equipped with Yokogawa CSUX1-spinning disc, Photometrics Evolve camera, and Metamorph software) using 491 and 561 nm laser lines and a GFP filter (495–560 nm).

### RNA isolation and gene expression analyses

Treated MsK8 cell samples were freeze dried and ground in 1 mL of TRIzol reagent (Invitrogen). Total RNA was extracted with a NucleoSpin RNA Plant kit (Macherey-Nagel Inc.) according to manufacturer’s instructions. cDNA was synthesized from 1 μg of total RNA and was reverse transcribed using oligo(dT) primers and M-MLV reverse transcriptase (Promega). Quantitative RT-PCR was performed using an ABI7300 real-time PCR system (Applied Biosystems). Expression of *Phytophthora* or tomato actin genes was used as internal control (Additional file 6: Table S3). Primers used are listed in Additional file 10: Table S4. Data were analyzed using ABI 7300 SDS 1.3.1.21 software and the comparative C_T_ (also referred as $2^{-{\Delta\Delta\text{C}_{\text{T}}}}$) method [49].

### Electrolyte leakage measurements

Electrolyte leakage was measured on 1 mL samples of treated MsK8 cells. To each sample, 4 mL of water was added and incubated for 2 h at RT with gentle agitation. Electrolyte leakage was determined by measuring conductivity (mS/cm) using a digital conductivity meter equipped with LabX direct PH 2.1 software (Mettler Toledo). Statistical analysis of the data was conducted by one-way ANOVA (P < 0.05) with IBM SPSS statistics 19 software.

### ROS measurements

ROS production was determined by measuring H_2_O_2_ using a modified xylenol orange assay [50, 51]. Xylenol orange reagent was freshly prepared according to Choi et al. [51]. In particular, 100 μl of treated cells were added to 500 μl 0.2 N HCl and mixed by vortexing for 30 s. Subsequently, samples were centrifuged for 5 min at 12.000 rpm. Supernatants were collected and mixed with 233 μl 50 mM phosphate buffer (pH 5.7) and 467 μl 0.2 N NaOH. Subsequently, 100 μl were mixed with 1 mL of fresh-prepared xylenol orange reagent and incubated for 30 min at RT in the dark. Absorbance was measured at 560 nm using a NanoDrop 1000 Spectrophotometer v3.7 (Thermo Scientific). To determine the H_2_O_2_ concentrations in the MsK8 samples, in each measurement a standard curve was made from absorbance values of samples with increasing concentrations of H_2_O_2_.

### Elicitor treatments

For elicitation assays, 2.5 mL of MsK8 or BY-2 cells were incubated for 1 h at RT with gentle shaking. Subsequently, cells were treated with *P.* *infestans* recombinant INF1 or INF2B elicitins purified from *E.* *coli*, or the bacterial MAMP peptide flg22 (Genscript), at different concentrations. Responsiveness of cell suspensions was measured as a pH shift by using a digital pH meter equipped with LabX direct PH 2.1 software (Mettler Toledo). Data were collected up to 30 min after elicitor treatment. MQ water was used as a negative control treatment.

## Additional files

**Additional file 1: Table S1.** *Phytophthora* isolates used in this study.

**Additional file 2: Figure S1.** MsK8 cells infected with different *Phytophthora* spp. MsK8 cells were inoculated with (A) *P.* *infestans* 14-3-GFP, (B) *P.* *capsici* LT51 and (C) *P.* *palmivora* GFP3. Bright field images showing primary infection at 6 hpi, secondary infection at 16 hpi, and sporangia formation at 48 hpi. Arrows point to the sites of penetration (left panels) or sporangia (right panels). Bars represent 100 μm.

**Additional file 3: Table S2.** Infection efficiency of *Phytophthora infestans* strain 14-3-GFP on MsK8 cells. The efficiency was quantified by determining the percentage of infected cells at 16 h post inoculation. For each sample a total of 500 cells was monitored in triplicate.

**Additional file 4: Figure S2.** Electrolyte leakage of MsK8 cells (A) upon inoculation with *Phytophthora* zoospores (zsp) or (B) treatment with zoospore exudate (ZE) measured as conductivity at various time points. Colors of the bars represent a specific species and/or strain as indicated and correspond to the colors in Fig. 2. Error bars represent standard deviation (n = 3).

**Additional file 5: Figure S3.** Responsiveness of MsK8 and BY-2 cells to *P.* *infestans* elicitins. MsK8 cells (A) and BY-2 cells (B), treated with *P.* *infestans* elicitins INF1 and INF2B. MsK8 cells treated with *P.* *infestans* elicitins INF1 and INF2B and flg22 (C). pH values were measured every 3 s during 20 min. ΔpH max value is the difference between the highest and the lowest pH value measured within 15 min after treatment. Error bars represent standard deviation (n = 3).

**Additional file 6: Table S3.** Genes selected for expression analysis by qRT-PCR.

**Additional file 7: Figure S4.** Expression of *P. infestans* genes upon inoculation of MsK8 cells with *P.* *infestans* 14-3-GFP (A), IPO-C (B) and T20-2 (C). Expression of stage-specific genes *HMP1*, *NPP1* and *CDC14, IPI*-*B* and various RXLR effector genes upon inoculation of MsK8 cells with zoospores. Expression levels were determined by qRT-PCR and the values at each time point were calculated relative to the expression level at time point 0 (0 hpi). Expression of the actin gene *ActA* was used as endogenous control.

**Additional file 8: Figure S5.** Expression of defense marker genes upon (A) inoculation of MsK8 cells with zoospores (zsp) or(B) treatment with zoospore exudate (ZE) of *P.* *infestans* strains IPO-C and T20-2. Defense genes include genes encoding pathogenesis-related proteins (PR), chitinases (Chi), a hypersensitivity marker (HSR203J) and isoforms of the subtilase P69 (P69a/b and P69c). Expression levels were determined by qRT-PCR and the values were calculated relative to the expression level at time point 0 (0 hpi). Expression of the tomato *ActA* was used as endogenous control.

**Additional file 9: Figure S6.** Expression profiling of tomato defense marker genes upon treatment of MsK8 cells with ZE of *P.* *infestans* 14-3-GFP (Pi), *P.* *sojae* P6497 (Ps), *P.* *capsici* LT263 (Pc) and *P.* *palmivora* GFP3 (Pp). Defense genes include genes encoding pathogenesis-related proteins (PR), chitinases (Chi), a hypersensitivity marker (HSR203J) and isoforms of the subtilase P69 (P69a/b and P69c). Expression levels were determined by qRT-PCR and the values were calculated relative to the expression level at time point 0 (0 hpi). Expression of the tomato *ActA* was used as endogenous control.

**Additional file 10: Table S4.** qRT-PCR primers used in this study.

## References

[1] Tyler BM, Rouxel T, Sessa G (2012). Effectors of fungi and oomycetes: their virulence and avirulence functions and translocation from pathogen to host cells. Molecular plant immunity.

[2] Wirthmueller L, Maqbool A, Banfield MJ (2013). On the front line: structural insights into plant-pathogen interactions. Nat Rev Microbiol.

[3] Stam R, Mantelin S, McLellan H, Thilliez G (2014). The role of effectors in nonhost resistance to filamentous plant pathogens. Front Plant Sci.

[4] Mott GA, Middleton MA, Desveaux D, Guttman DS (2014). Peptides and small molecules of the plant-pathogen apoplastic arena. Front Plant Sci.

[5] Dodds PN, Rathjen JP (2010). Plant immunity: towards an integrated view of plant–pathogen interactions. Nat Rev Genet.

[6] Kroon LPNM, Brouwer H, de Cock AWAM, Govers F (2011). The Genus *Phytophthora* Anno 2012. Phytopathology.

[7] Kamoun S, Furzer O, Jones JDG, Judelson HS, Ali GS, Dalio RJD, Roy SG, Schena L, Zambounis A, Panabières F (2015). The Top 10 oomycete pathogens in molecular plant pathology. Mol Plant Pathol.

[8] Petre B, Kamoun S (2014). How do filamentous pathogens deliver effector proteins into plant cells?. PLoS Biol.

[9] Whisson SC, Boevink PC, Wang S, Birch PRJ (2016). The cell biology of late blight disease. Curr Opin Microbiol.

[10] Oh SK, Kamoun S, Choi D (2010). Oomycetes RXLR effectors function as both activator and suppressor of plant immunity. Plant Pathol J.

[11] Oliva R, Win J, Raffaele S, Boutemy L, Bozkurt TO, Chaparro-Garcia A, Segretin ME, Stam R, Schornack S, Cano LM (2010). Recent developments in effector biology of filamentous plant pathogens. Cell Microbiol.

[12] Kamoun S, van West P, Vleeshouwers VG, de Groot KE, Govers F (1998). Resistance of *Nicotiana benthamiana* to *Phytophthora infestans* is mediated by the recognition of the elicitor protein INF1. Plant Cell..

[13] van West P, de Jong AJ, Judelson HS, Emons AMC, Govers F (1998). The ipiO gene of *Phytophthora infestans* is highly expressed in invading hyphae during infection. Fungal Genet Biol.

[14] Kato K, Matsoumoto T, Koiwai S, Mizusaki S, Nishida K, Nogushi M, Tamaki E, Terui G (1972). Liquid suspension culture of tobaco cells. Ferment technology today.

[15] Able AJ, Guest DI, Sutherland MW (2000). Hydrogen peroxide yields during the incompatible interaction of tobacco suspension cells inoculated with *Phytophthora nicotianae*. Plant Physiol.

[16] Able AJ, Guest DI, Sutherland MW (2001). Relationship between transmembrane ion movements, production of reactive oxygen species and the hypersensitive response during the challenge of tobacco suspension cells by zoospores of *Phytophthora nicotianae*. Physiol Mol Plant Pathol.

[17] Baz M, Tran D, Kettani-Halabi M, Samri SE, Jamjari A, Biligui B, Meimoun P, El-Maarouf-Bouteau H, Garmier M, Saindrenan P (2012). Calcium- and ROS-mediated defence responses in BY2 tobacco cells by nonpathogenic *Streptomyces* sp.. J Appl Microbiol.

[18] Ruelland E, Pokotylo I, Cantrel C, Djafi N, Repellin A, Zachowski A (2014). Salicylic acid modulates levels of phosphoinositide dependent-phospholipase C substrates and products to remodel the Arabidopsis suspension cell transcriptome. Front Plant Sci.

[19] Ampofo-Asiama J, Baiye VMM, Hertog MLATM, Waelkens E, Geeraerd AH, Nicolai BM (2014). The metabolic response of cultured tomato cells to low oxygen stress. Plant Biol.

[20] Aimé S, Cordier C, Alabouvette C, Olivain C (2008). Comparative analysis of PR gene expression in tomato inoculated with virulent *Fusarium oxysporum* f. sp. *lycopersici* and the biocontrol strain *F. oxysporum* Fo47. Physiol Mol. Plant Pathol.

[21] Takahashi H, Matsumura H, Kawai-Yamada M, Uchimiya H (2008). The cell death factor, cell wall elicitor of rice blast fungus (*Magnaporthe grisea*) causes metabolic alterations including GABA shunt in rice cultured cells. Plant Signal Behav.

[22] Naton B, Hahlbrock K, Schmelzer E (1996). Correlation of rapid cell death with metabolic changes in fungus-infected, cultured parsley cells. Plant Physiol.

[23] Val F, Desender S, Bernard K, Potin P, Hamelin G, Andrivon D (2008). A culture filtrate of *Phytophthora infestans* primes defense reaction in potato cell suspensions. Phytopathology.

[24] Moroz N, Fritch KR, Marcec MJ, Tripathi D, Smertenko A, Tanaka K (2017). Extracellular alkalinization as a defense response in potato cells. Front Plant Sci.

[25] Nakazawa-Ueji YE, Núñez-Pastrana R, Souza-Perera RA, Santana-Buzzy N, Zúñiga-Aguilar JJ (2009). Mycelium homogenates from a virulent strain of *Phytophthora capsici* promote a defence-related response in cell suspensions from *Capsicum chinense*. Eur J Plant Pathol.

[26] Koornneef M, Hanhart CJ, Martinelli L (1987). A genetic analysis of cell culture traits in tomato. Theor Appl Genet.

[27] Sánchez-Vallet A, Saleem-Batcha R, Kombrink A, Hansen G, Valkenburg D-J, Thomma BPHJ, Mesters JR (2013). Fungal effector Ecp6 outcompetes host immune receptor for chitin binding through intrachain LysM dimerization. eLife.

[28] Felix G, Duran JD, Volko S, Boller T (1999). Plants have a sensitive perception system for the most conserved domain of bacterial flagellin. Plant J.

[29] Felix G, Regenass M, Boller T (1993). Specific perception of subnanomolar concentrations of chitin fragments by tomato cells: induction of extracellular alkalinization, changes in protein phosphorylation, and establishment of a refractory state. Plant J.

[30] Zuluaga AP, Vega-Arreguín JC, Fei Z, Ponnala L, Lee SJ, Matas AJ, Patev S, Fry WE, Rose JKC (2016). Transcriptional dynamics of *Phytophthora infestans* during sequential stages of hemibiotrophic infection of tomato. Mol Plant Pathol.

[31] Wang Y, Weide R, Govers F, Bouwmeester K (2015). L-type lectin receptor kinases in *Nicotiana benthamiana* and tomato and their role in *Phytophthora* resistance. J Exp Bot.

[32] Akinrefon OA (1969). Biochemical studies on plant tissues infected by *Phytophthora palmivora* (Butl.). Ann Appl Biol.

[33] Le Berre J-Y, Engler G, Panabières F (2008). Exploration of the late stages of the tomato–*Phytophthora parasitica* interactions through histological analysis and generation of expressed sequence tags. New Phytol.

[34] Torres MA, Jones JDG, Dangl JL (2006). Reactive oxygen species signaling in response to pathogens. Plant Physiol.

[35] Meng Y, Zhang Q, Ding W, Shan W (2014). *Phytophthora parasitica*: a model oomycete plant pathogen. Mycology.

[36] Kebdani N, Pieuchot L, Deleury E, Panabières F, Le Berre JY, Gourgues M (2010). Cellular and molecular characterization of *Phytophthora parasitica* appressorium-mediated penetration. New Phytol.

[37] Felix G, Grosskopf DG, Regenass M, Basse CW, Boller T (1991). Elicitor-induced ethylene biosynthesis in tomato cells: characterization and use as a bioassay for elicitor action. Plant Physiol.

[38] Jiang RHY, Tyler BM, Whisson SC, Hardham AR, Govers F (2006). Ancient origin of elicitin gene clusters in Phytophthora genomes. Mol Biol Evol.

[39] Du J, Verzaux E, Chaparro-Garcia A, Bijsterbosch G, Keizer LCP, Zhou J, Liebrand TWH, Xie C, Govers F, Robatzek S (2015). Elicitin recognition confers enhanced resistance to *Phytophthora infestans* in potato. Nat Plants.

[40] Jupe J, Stam R, Howden A, Morris J, Zhang R, Hedley P, Huitema E (2013). *Phytophthora capsici*-tomato interaction features dramatic shifts in gene expression associated with a hemi-biotrophic lifestyle. Genome Biol.

[41] Avrova AO, Boevink PC, Young V, Grenville-Briggs LJ, Van West P, Birch PRJ, Whisson SC (2008). A novel *Phytophthora infestans* haustorium-specific membrane protein is required for infection of potato. Cell Microbiol.

[42] de Vries S, von Dahlen JK, Uhlmann C, Schnake A, Kloesges T, Rose LE (2016). Signatures of selection and host-adapted gene expression of the *Phytophthora infestans* RNA silencing suppressor PSR2. Mol Plant Pathol.

[43] Abrahamian M, Ah-Fong AM, Davis C, Andreeva K, Judelson HS (2016). Gene expression and silencing studies in *Phytophthora infestans* reveal infection-specific nutrient transporters and a role for the nitrate reductase pathway in plant pathogenesis. PLoS Pathog.

[44] Ah Fong AMV, Judelson HS (2003). Cell cycle regulator Cdc14 is expressed during sporulation but not hyphal growth in the fungus-like oomycete *Phytophthora infestans*. Mol Microbiol.

[45] Jiang RHY, Tripathy S, Govers F, Tyler BM (2008). RXLR effector reservoir in two *Phytophthora* species is dominated by a single rapidly evolving superfamily with more than 700 members. Proc Natl Acad Sci.

[46] Vleeshouwers VGAA, Raffaele S, Vossen JH, Champouret N, Oliva R, Segretin ME, Rietman H, Cano LM, Lokossou A, Kessel G (2011). Understanding and exploiting late blight resistance in the age of effectors. Annu Rev Phytopathol.

[47] Gilroy EM, Breen S, Whisson SC, Squires J, Hein I, Kaczmarek M, Turnbull D, Boevink PC, Lokossou A, Cano LM (2011). Presence/absence, differential expression and sequence polymorphisms between PiAVR2 and PiAVR2-like in *Phytophthora infestans* determine virulence on R2 plants. New Phytol.

[48] Wang Q, Han C, Ferreira AO, Yu X, Ye W, Tripathy S, Kale SD, Gu B, Sheng Y, Sui Y (2011). Transcriptional programming and functional interactions within the *Phytophthora sojae* RXLR effector repertoire. Plant Cell.

[49] Schmittgen TD, Livak KJ (2008). Analyzing real-time PCR data by the comparative CT method. Nat Protoc.

[50] Gay C, Collins J, Gebicki JM (1999). Hydroperoxide assay with the ferric–xylenol orange complex. Anal Biochem.

[51] Choi HW, Kim YJ, Lee SC, Hong JK, Hwang BK (2007). Hydrogen peroxide generation by the pepper extracellular peroxidase CaPO2 activates local and systemic cell death and defense response to bacterial pathogens. Plant Physiol.

