# Supplementary material for: Infection of a tomato cell culture by Phytophthora infestans; a versatile tool to study Phytophthora-host interactions
Source: Plant Methods. 2017 Oct 25;13:88. doi: 10.1186/s13007-017-0240-0 (PMC5657071; doi:10.1186/s13007-017-0240-0)
Supplement: Supplementary file 1 — Additional file 1: Table S1. Phytophthora isolates used in this study. [file 13007_2017_240_MOESM1_ESM.docx]

**Table S1.** *Phytophthora* isolates used in this study*.*

| ***Phytophthora***  **species** | **Isolate** | **Origin** | | | **References and remarks** |
| --- | --- | --- | --- | --- | --- |
|  |  | Country | Year | Source |  |
| ***P. infestans*** | IPO-C | Belgium | 1982 | Potato | - |
|  | T20-2 (H20P02) | The Netherlands | 1993 | # | F1 progeny from a cross between *P. infestans* 80029 and 88133 (Drenth *et al.*, 1994) |
|  | 14-3-GFP | # | # | Potato | *GFP*-expressing transformant of *P. infestans* H30P02 |
| ***P. capsici*** | LT263 | USA | 2004 | Pumpkin | (Donahoo & Lamour, 2008) |
|  | LT3239 | USA | 2006 | Pumpkin | (Wang *et al.*, 2013) |
|  | LT51 | USA | 1997 | Cucumber | - |
| ***P. palmivora*** | GFP3 | # | # | # | *GFP-*expressing transformant of *P. palmivora* P6390 (Vijn & Govers, 2003) |
| ***P. sojae*** | P6497 | USA | 2001 | Soybean | (Förster *et al.*, 1994) |
| ***P. parasitica*** | H1111 | Australia | ## | Tobacco | (Robold & Hardham, 1998) |

# not applicable; ## unknown
