# Supplementary figures and images for: Infection of a tomato cell culture by Phytophthora infestans; a versatile tool to study Phytophthora-host interactions
Source: Plant Methods. 2017 Oct 25;13:88. doi: 10.1186/s13007-017-0240-0 (PMC5657071; doi:10.1186/s13007-017-0240-0)

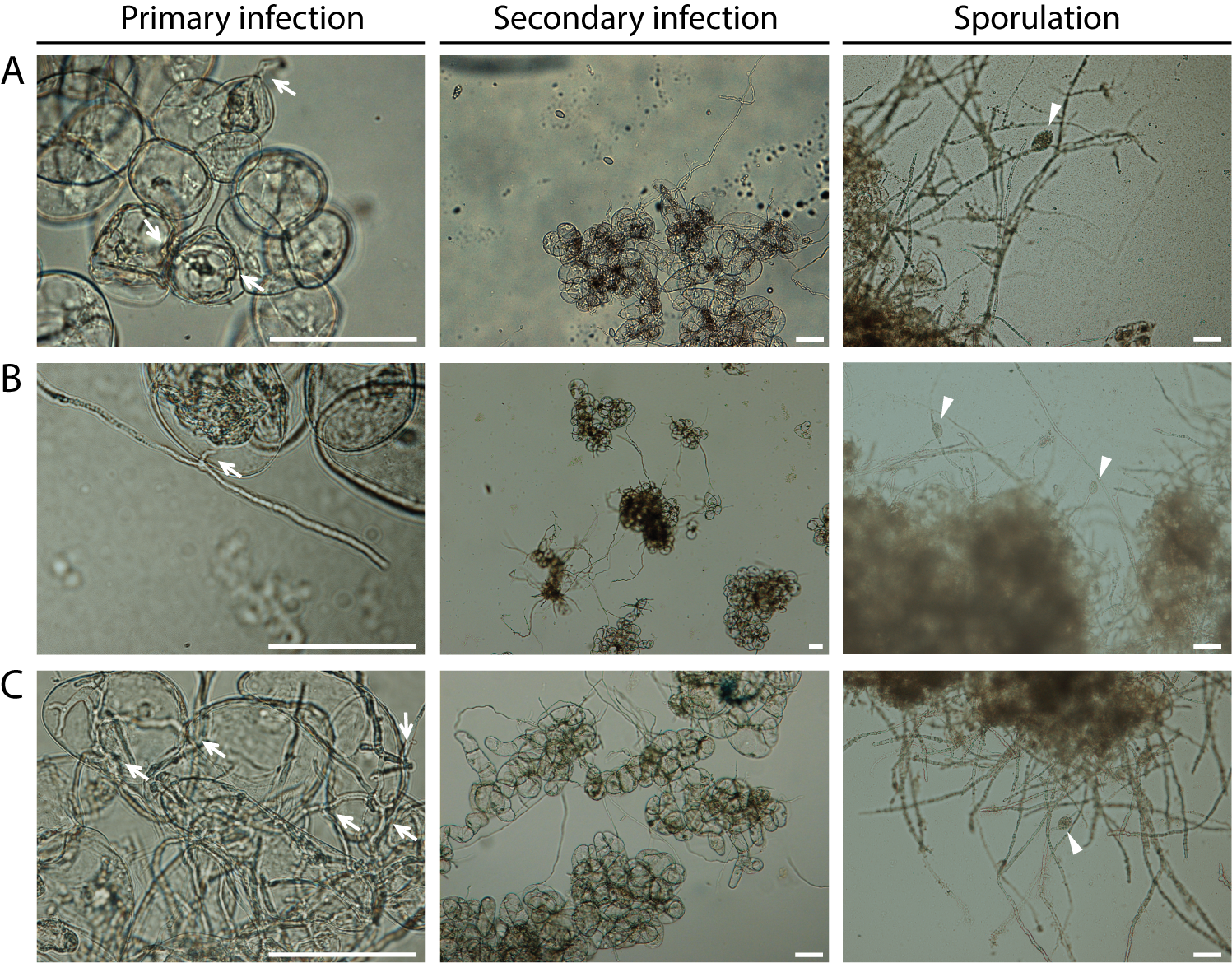

Supplement: Supplementary file 2 — Additional file 2: Figure S1. MsK8 cells infected with different Phytophthora spp. MsK8 cells were inoculated with (A) P. infestans 14-3-GFP, (B) P. capsici LT51 and (C) P. palmivora GFP3. Bright field images showing primary infection at 6 hpi, secondary infection at 16 hpi, and sporangia formation at 48 hpi. Arrows point to the sites of penetration (left panels) or sporangia (right panels). Bars represent 100 μm. [file 13007_2017_240_MOESM2_ESM.tif]

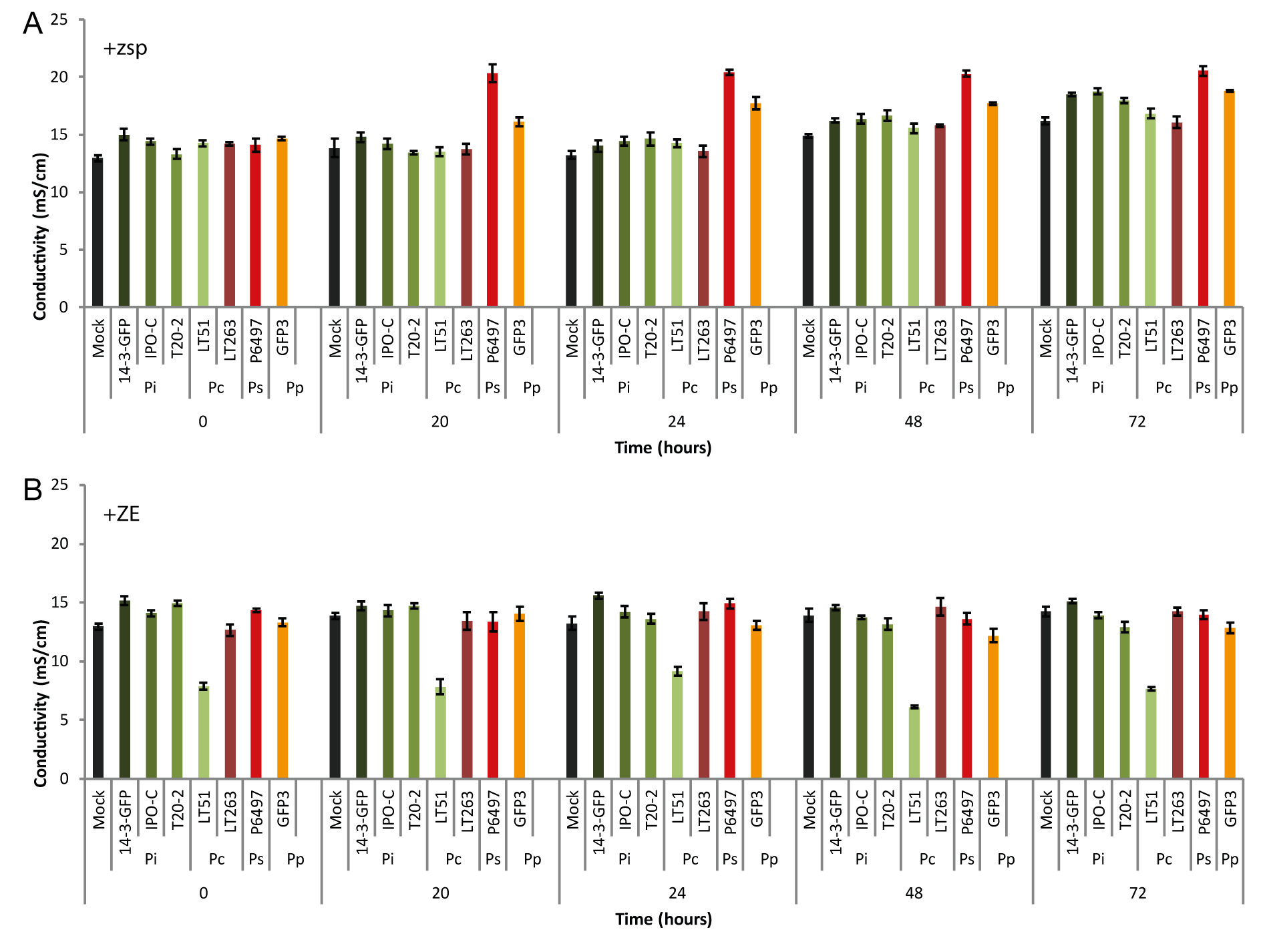

Supplement: Supplementary file 4 — Additional file 4: Figure S2. Electrolyte leakage of MsK8 cells (A) upon inoculation with Phytophthora zoospores (zsp) or (B) treatment with zoospore exudate (ZE) measured as conductivity at various time points. Colors of the bars represent a specific species and/or strain as indicated and correspond to the colors in Fig. 2. Error bars represent standard deviation (n = 3). [file 13007_2017_240_MOESM4_ESM.tif]

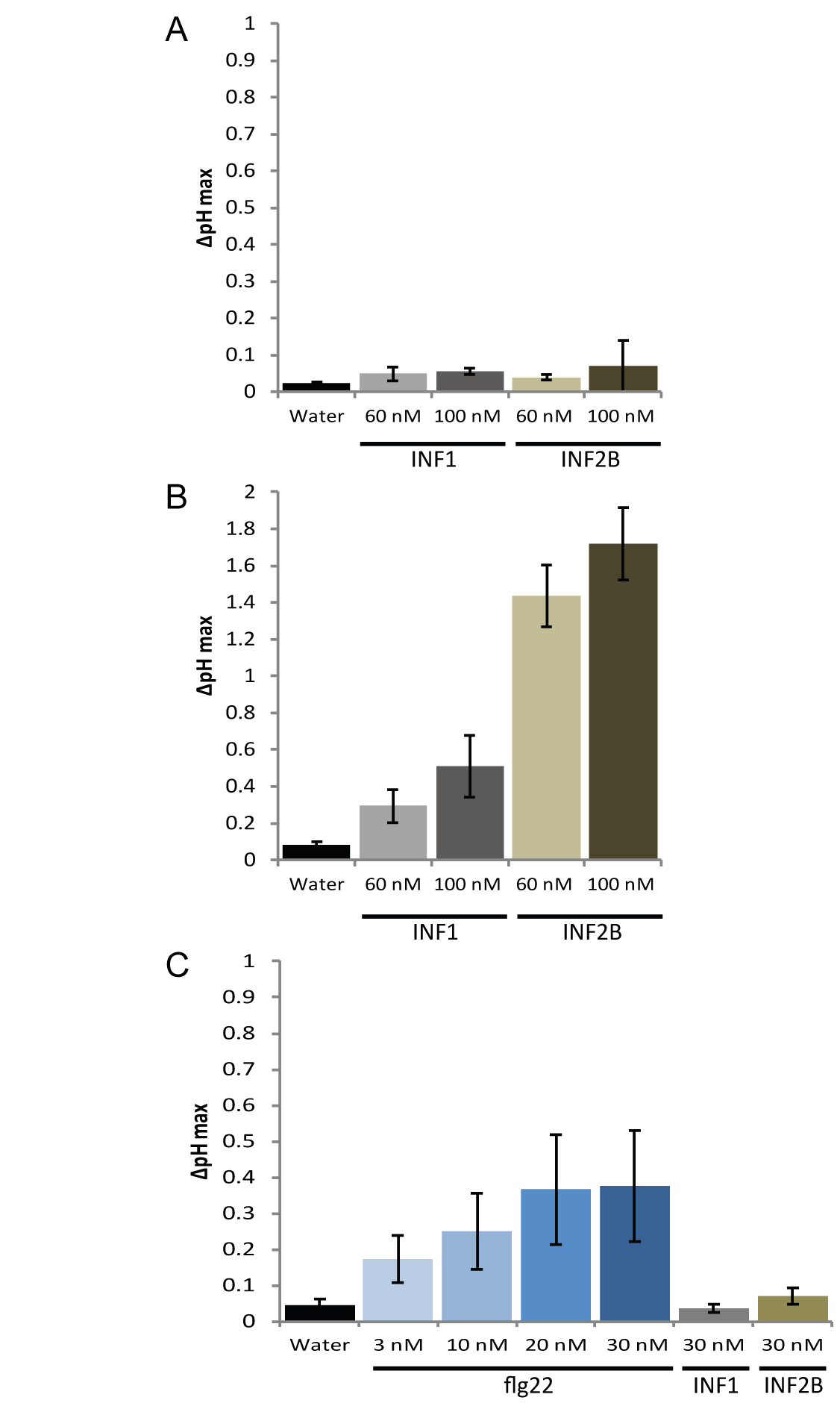

Supplement: Supplementary file 5 — Additional file 5: Figure S3. Responsiveness of MsK8 and BY-2 cells to P. infestans elicitins. MsK8 cells (A) and BY-2 cells (B), treated with P. infestans elicitins INF1 and INF2B. MsK8 cells treated with P. infestans elicitins INF1 and INF2B and flg22 (C). pH values were measured every 3 s during 20 min. ΔpH max value is the difference between the highest and the lowest pH value measured within 15 min after treatment. Error bars represent standard deviation (n = 3). [file 13007_2017_240_MOESM5_ESM.tif]

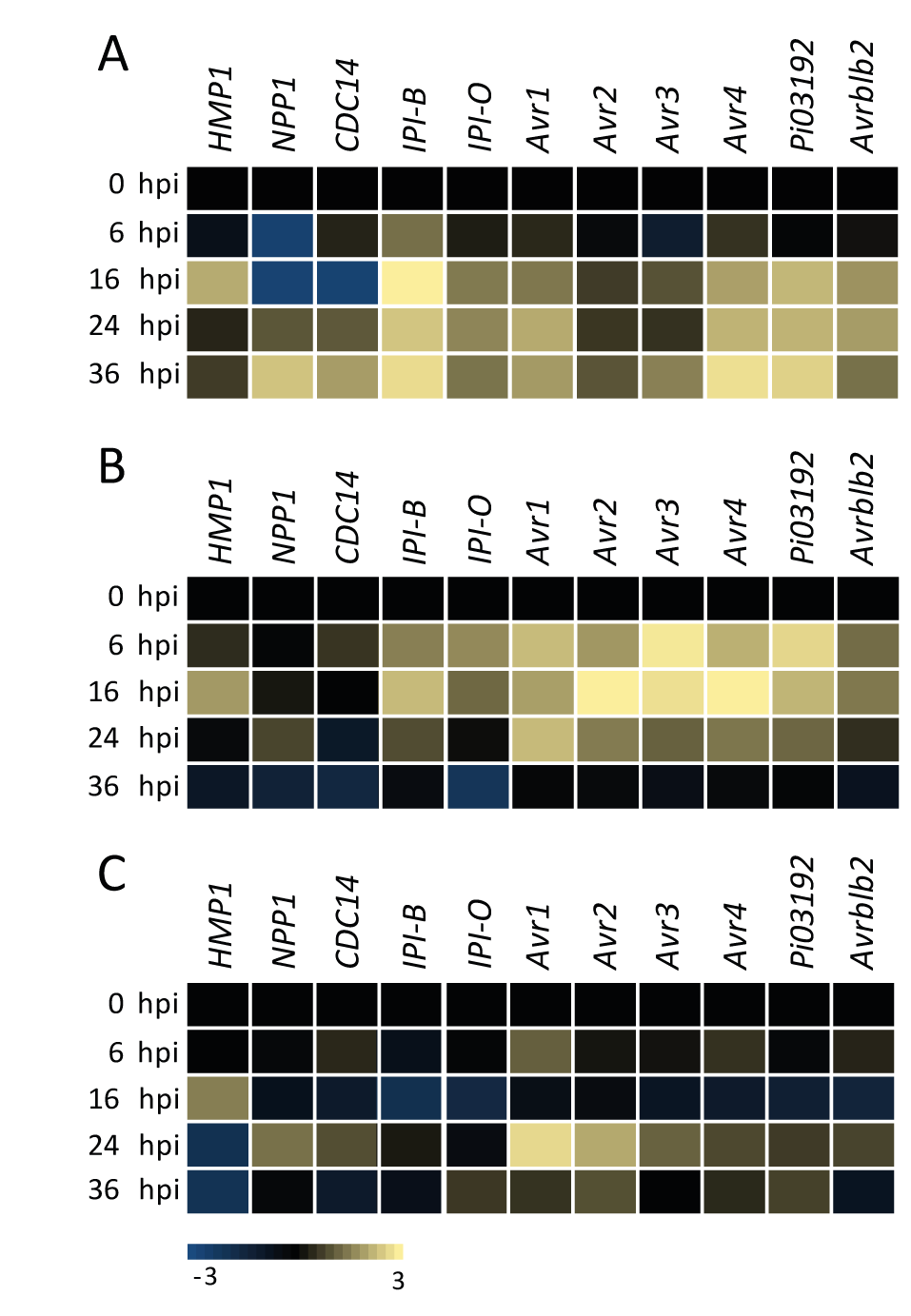

Supplement: Supplementary file 7 — Additional file 7: Figure S4. Expression of P. infestans genes upon inoculation of MsK8 cells with P. infestans 14-3-GFP (A), IPO-C (B) and T20-2 (C). Expression of stage-specific genes HMP1, NPP1 and CDC14, IPI-B and various RXLR effector genes upon inoculation of MsK8 cells with zoospores. Expression levels were determined by qRT-PCR and the values at each time point were calculated relative to the expression level at time point 0 (0 hpi). Expression of the actin gene ActA was used as endogenous control. [file 13007_2017_240_MOESM7_ESM.tif]

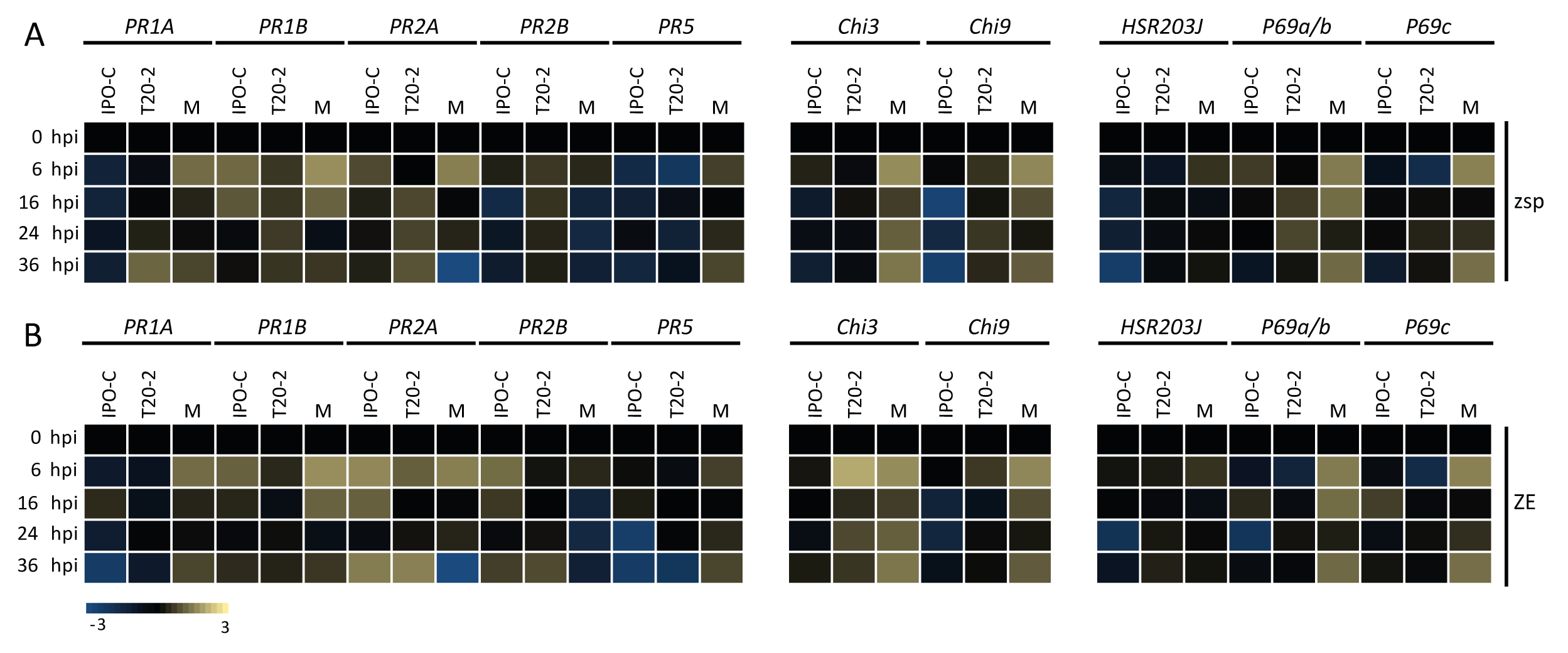

Supplement: Supplementary file 8 — Additional file 8: Figure S5. Expression of defense marker genes upon (A) inoculation of MsK8 cells with zoospores (zsp) or(B) treatment with zoospore exudate (ZE) of P. infestans strains IPO-C and T20-2. Defense genes include genes encoding pathogenesis-related proteins (PR), chitinases (Chi), a hypersensitivity marker (HSR203J) and isoforms of the subtilase P69 (P69a/b and P69c). Expression levels were determined by qRT-PCR and the values were calculated relative to the expression level at time point 0 (0 hpi). Expression of the tomato ActA was used as endogenous control. [file 13007_2017_240_MOESM8_ESM.tif]

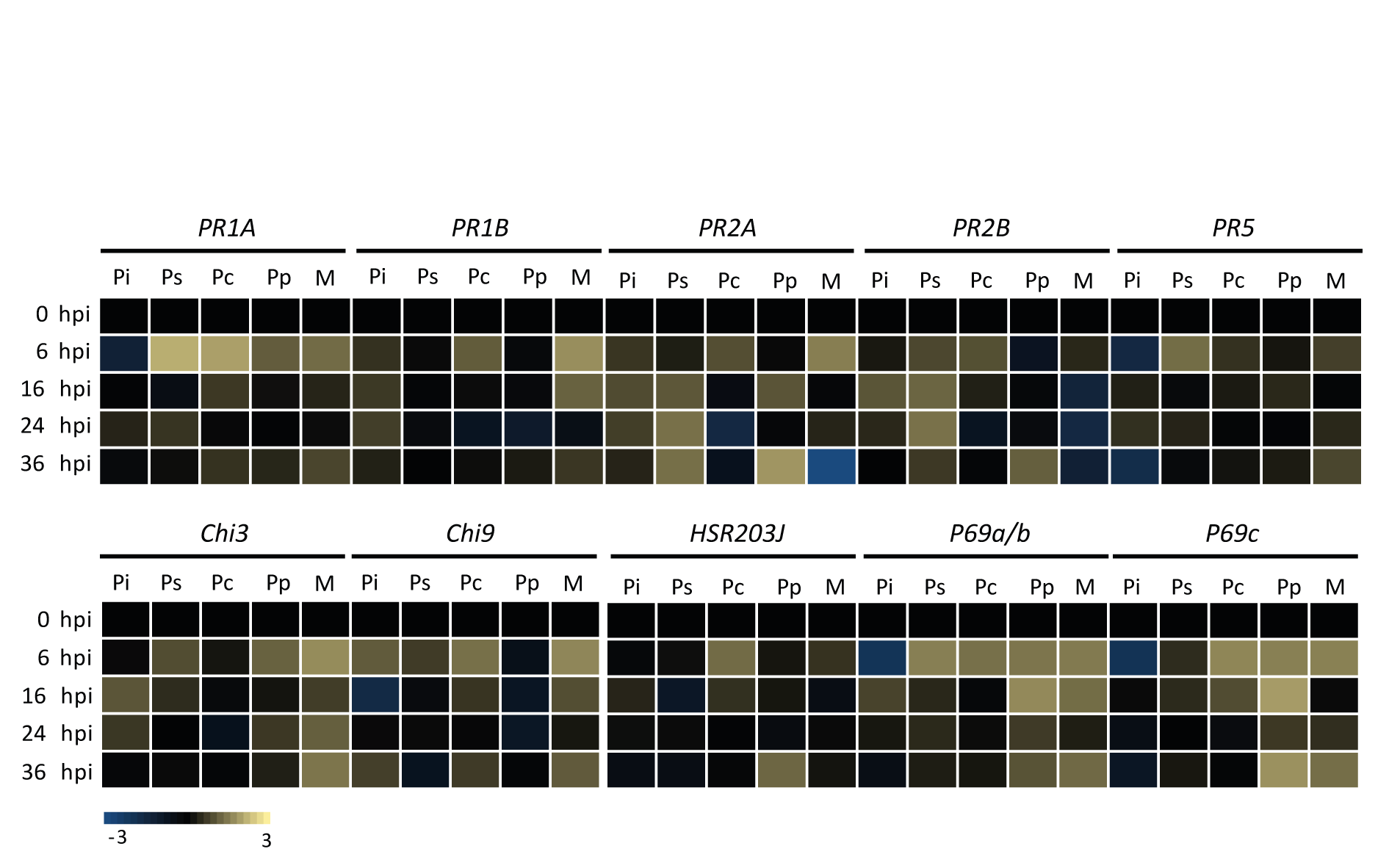

Supplement: Supplementary file 9 — Additional file 9: Figure S6. Expression profiling of tomato defense marker genes upon treatment of MsK8 cells with ZE of P. infestans 14-3-GFP (Pi), P. sojae P6497 (Ps), P. capsici LT263 (Pc) and P. palmivora GFP3 (Pp). Defense genes include genes encoding pathogenesis-related proteins (PR), chitinases (Chi), a hypersensitivity marker (HSR203J) and isoforms of the subtilase P69 (P69a/b and P69c). Expression levels were determined by qRT-PCR and the values were calculated relative to the expression level at time point 0 (0 hpi). Expression of the tomato ActA was used as endogenous control. [file 13007_2017_240_MOESM9_ESM.tif]
