# Supplementary material for: Infection of a tomato cell culture by Phytophthora infestans; a versatile tool to study Phytophthora-host interactions
Source: Plant Methods. 2017 Oct 25;13:88. doi: 10.1186/s13007-017-0240-0 (PMC5657071; doi:10.1186/s13007-017-0240-0)
Supplement: Supplementary file 3 — Additional file 3: Table S2. Infection efficiency of Phytophthora infestans strain 14-3-GFP on MsK8 cells. The efficiency was quantified by determining the percentage of infected cells at 16 h post inoculation. For each sample a total of 500 cells was monitored in triplicate. [file 13007_2017_240_MOESM3_ESM.docx]

**Table S2.** Infection efficiency of *Phytophthora* *infestans* strain 14-3-GFP on MsK8 cells. The efficiency was quantified by determining the percentage of infected cells at 16 hours post inoculation. For each sample a total of 500 cells was monitored in triplicate.

| **%** | | **Inoculum concentration (zoospores/ml)** | | | | |
| --- | --- | --- | --- | --- | --- | --- |
|  |  | 10^4^ | 5x10^4^ | 10^5^ | 5x10^5^ | 10^6^ |
| **Infected MsK8 cells^a^** | | 17.0 ± 2.8 | 18.8 ± 3.9 | 36.4 ± 1.2 | 32.0 ± 2.8 | 30.4 ± 3.4 |
|  | **Primary infection^b^** | 81.1 ± 3.4 | 80.0 ± 4.2 | 73.6 ± 3.6 | 78.7 ± 5.7 | 74.3 ± 2.4 |
|  | **Secondary infection^c^** | 18.9 ± 2.1 | 20.0 ± 2.2 | 26.4 ± 1.4 | 21.2 ± 3.3 | 25.7 ± 1.4 |
|  | **Cells containing haustoria^d^** | 12.9 ± 1.8 | 18.1 ± 3.1 | 21.4 ± 1.1 | 18.1 ± 2.1 | 17.1 ± 2.0 |

^a^ Cells that had been penetrated by *Phytophthora* were counted as infected.

^b^ Infected cells due to primary infection i.e. penetrated by germ tubes emerging from cysts.

^c^ Infected cells due to secondary infection i.e. penetrated by hyphae expanding from a neighbouring infected cell.

^d^ Infected cells containing haustoria.
