# Supplementary material for: Infection of a tomato cell culture by Phytophthora infestans; a versatile tool to study Phytophthora-host interactions
Source: Plant Methods. 2017 Oct 25;13:88. doi: 10.1186/s13007-017-0240-0 (PMC5657071; doi:10.1186/s13007-017-0240-0)
Supplement: Supplementary file 6 — Additional file 6: Table S3. Genes selected for expression analysis by qRT-PCR. [file 13007_2017_240_MOESM6_ESM.docx]

**Table S3.** Genes selected for expression analysis by qRT-PCR.

| Organism | Gene | Function | Reference |
| --- | --- | --- | --- |
|  | *ActA* | Structural protein |  |
| *Phytophthora* spp. | *HMP1* | Haustorial membrane protein | (Avrova *et al.*, 2008) |
|  | *NPP1* | Necrosis-inducing protein | (Qutob *et al.*, 2002) |
|  | *CDC1*4 | Cell cycle regulator | (Ah Fong & Judelson, 2003) |
|  | *IPI-B* | Putative cell wall protein | (Pieterse *et al.*, 1994) |
|  | *IPI-O* | RXLR effector | (van West *et al.*, 1998) |
|  | *Avr1* | RXLR effector | (Guo, 2008) |
|  | *Avr2* | RXLR effector | (Gilroy *et al.*, 2011) |
|  | *Avr3* | RXLR effector | (Armstrong *et al.*, 2005) |
|  | *Avr4* | RXLR effector | (van Poppel *et al.*, 2008) |
|  | *Avrblb2* | RXLR effector | (Oh *et al.*, 2009) |
|  | *PITG_03192 (RD28)* | RXLR effector | (McLellan *et al.*, 2013, Oh et al., 2009) |
| *Solanum lycopersicum* | *ActA* | Structural protein |  |
|  | *Chi3* | Chitinase | (Danhash *et al.*, 1993) |
|  | *Chi9* | Chitinase | (Danhash et al., 1993) |
|  | *PR1a* | Pathogenesis-related protein | (van Kan *et al.*, 1992) |
|  | *PR1b* | Pathogenesis-related protein | (van Kan et al., 1992) |
|  | *PR2a (GlucA)* | Glucanase | (van Kan et al., 1992) |
|  | *PR2b (GlucB)* | Beta 1,3-glucanase | (van Kan et al., 1992) |
|  | *PR5* | Pathogenesis-related protein | (Singh *et al.*, 1987, Rodrigo *et al.*, 1993, Anžlovar & Dermastia, 2003) |
|  | *HSR203J* | Hypersensitive response marker | (Pontier *et al.*, 1998) |
|  | *P69 (a, b, c)* | Subtilase | (Meichtry *et al.*, 1999) |

**References**

**Ah Fong, A.M.V. and Judelson, H.S.** (2003) Cell cycle regulator Cdc14 is expressed during sporulation but not hyphal growth in the fungus-like oomycete *Phytophthora infestans*. *Mol. Microbiol.,* **50,** 487-494.

**Anžlovar, S. and Dermastia, M.** (2003) The comparative analysis of osmotins and osmotin-like PR-5 proteins. *Plant Biol.,* **5,** 116-124.

**Armstrong, M.R., Whisson, S.C., Pritchard, L., Bos, J.I.B., Venter, E., Avrova, A.O., Rehmany, A.P., Böhme, U., Brooks, K., Cherevach, I., Hamlin, N., White, B., Fraser, A., Lord, A., Quail, M.A., Churcher, C., Hall, N., Berriman, M., Huang, S., Kamoun, S., Beynon, J.L. and Birch, P.R.J.** (2005) An ancestral oomycete locus contains late blight avirulence gene Avr3a, encoding a protein that is recognized in the host cytoplasm. *Proc. Natl. Acad. Sci.,* **102,** 7766-7771.

**Avrova, A.O., Boevink, P.C., Young, V., Grenville-Briggs, L.J., Van West, P., Birch, P.R.J. and Whisson, S.C.** (2008) A novel *Phytophthora infestans* haustorium-specific membrane protein is required for infection of potato. *Cell. microbiol.,* **10,** 2271-2284.

**Danhash, N., Wagemakers, C.A.M., van Kan, J.A.L. and de Wit, P.J.G.M.** (1993) Molecular characterization of four chitinase cDNAs obtained from *Cladosporium fulvum*-infected tomato. *Plant Mol. Biol.,* **22,** 1017-1029.

**Donahoo, R.S. and Lamour, K.H.** (2008) Interspecific hybridization and apomixis between *Phytophthora capsici* and *Phytophthora tropicalis*. *Mycologia,* **100,** 911-920.

**Drenth, A., Tas, I.Q. and Govers, F.** (1994) DNA fingerprinting uncovers a new sexually reproducing population of *Phytophthora infestans* in the Netherlands. *Eur. J. Plant Pathol.,* **100,** 97-107.

**Förster, H., Tyler, B.M. and Coffey, M.D.** (1994) *Phytophthora sojae* races have arisen by clonal evolution and by rare outcrosses. *Mol. Plant-Microbe Interact.,* **7,** 780-791.

**Gilroy, E.M., Breen, S., Whisson, S.C., Squires, J., Hein, I., Kaczmarek, M., Turnbull, D., Boevink, P.C., Lokossou, A., Cano, L.M., Morales, J., Avrova, A.O., Pritchard, L., Randall, E., Lees, A., Govers, F., van West, P., Kamoun, S., Vleeshouwers, V.G.A.A., Cooke, D.E.L. and Birch, P.R.J.** (2011) Presence/absence, differential expression and sequence polymorphisms between PiAVR2 and PiAVR2-like in *Phytophthora infestans* determine virulence on R2 plants. *New Phytol.,* **191,** 763-776.

**Guo, J.** (2008) *Phytophthora infestans* avirulence genes: mapping, cloning and diversity in field isolates. Wageningen, the Netherlands: Wageningen university.

**McLellan, H., Boevink, P.C., Armstrong, M.R., Pritchard, L., Gomez, S., Morales, J., Whisson, S.C., Beynon, J.L. and Birch, P.R.J.** (2013) An RxLR effector from *Phytophthora infestans* prevents re-localisation of two plant NAC transcription factors from the endoplasmic reticulum to the nucleus. *PLoS Pathog,* **9,** e1003670.

**Meichtry, J., Amrhein, N. and Schaller, A.** (1999) Characterization of the subtilase gene family in tomato (*Lycopersicon esculentum* Mill.). *Plant Mol. Biol.,* **39,** 749-760.

**Oh, S.K., Young, C., Lee, M., Oliva, R., Bozkurt, T.O., Cano, L.M., Win, J., Bos, J.I.B., Liu, H.Y., van Damme, M., Morgan, W., Choi, D., Van der Vossen, E.A.G., Vleeshouwers, V.G.A.A. and Kamoun, S.** (2009) In planta expression screens of *Phytophthora infestans* RXLR effectors reveal diverse [henotypes, including activation of the *Solanum bulbocastanum* disease resistance protein Rpi-blb2. *The Plant Cell,* **21,** 2928-2947.

**Pieterse, C.M.J., Derksen, A.-M.C.E., Folders, J. and Govers, F.** (1994) Expression of the *Phytophthora infestans* ipiB and ipi0 genes in planta and in vitro. *Molecular and General Genetics MGG,* **244,** 269-277.

**Pontier, D., Tronchet, M., Rogowsky, P., Lam, E. and Roby, D.** (1998) Activation of hsr203, a plant gene expressed during incompatible plant-pathogen interactions, is correlated with programmed cell death. *Mol. Plant-Microbe Interact.,* **11,** 544-554.

**Qutob, D., Kamoun, S. and Gijzen, M.** (2002) Expression of a *Phytophthora sojae* necrosis-inducing protein occurs during transition from biotrophy to necrotrophy. *Plant J.,* **32,** 361-373.

**Robold, A.V. and Hardham, A.R.** (1998) Production of species-specific monoclonal antibodies that react with surface components on zoospores and cysts of *Phytophthora nicotianae*. *Canadian Journal of Microbiology,* **44,** 1161-1170.

**Rodrigo, I., Vera, P., Tornero, P., Hernandez-Yago, J. and Conejero, V.** (1993) cDNA cloning of viroid-induced tomato pathogenesis-related protein P23 (Characterization as a vacuolar antifungal factor). *Plant Physiol.,* **102,** 939-945.

**Singh, N.K., Bracker, C.A., Hasegawa, P.M., Handa, A.K., Buckel, S., Hermodson, M.A., Pfankoch, E., Regnier, F.E. and Bressan, R.A.** (1987) Characterization of Osmotin : A thaumatin-like protein associated with osmotic adaptation in plant cells. *Plant Physiol.,* **85,** 529-536.

**van Kan, J.A.L., Joosten, M.H.A.J., Wagemakers, C.A.M., van den Berg-Velthuis, G.C.M. and de Wit, P.J.G.M.** (1992) Differential accumulation of mRNAs encoding extracellular and intracellular PR proteins in tomato induced by virulent and avirulent races of *Cladosporium fulvum*. *Plant Mol. Biol.,* **20,** 513-527.

**van Poppel, P.M.J.A., Guo, J., van de Vondervoort, P.J.I., Jung, M.W.M., Birch, P.R.J., Whisson, S.C. and Govers, F.** (2008) The *Phytophthora infestans* avirulence gene Avr4 encodes an RXLR-dEER effector. *Mol. Plant-Microbe Interact.,* **21,** 1460-1470.

**van West, P., de Jong, A.J., Judelson, H.S., Emons, A.M.C. and Govers, F.** (1998) The ipiO gene of *Phytophthora infestans* is highly expressed in invading hyphae during infection. *Fungal Genet. Biol.,* **23,** 126-138.

**Vijn, I. and Govers, F.** (2003) *Agrobacterium tumefaciens* mediated transformation of the oomycete plant pathogen *Phytophthora infestans*. *Mol. Plant Pathol.,* **4,** 459-467.

**Wang, Y.A.N., Bouwmeester, K., van de Mortel, J.E., Shan, W. and Govers, F.** (2013) A novel Arabidopsis–oomycete pathosystem: differential interactions with *Phytophthora capsici* reveal a role for camalexin, indole glucosinolates and salicylic acid in defence. *Plant, Cell & Environment,* **36,** 1192-1203.
