# Supplementary material for: Infection of a tomato cell culture by Phytophthora infestans; a versatile tool to study Phytophthora-host interactions
Source: Plant Methods. 2017 Oct 25;13:88. doi: 10.1186/s13007-017-0240-0 (PMC5657071; doi:10.1186/s13007-017-0240-0)
Supplement: Supplementary file 10 — Additional file 10: Table S4. qRT-PCR primers used in this study. [file 13007_2017_240_MOESM10_ESM.docx]

**Table S4.** qRT-PCR primers used in this study.

|  | **Gene** | **Forward primer** | **Sequence (5’-3’)** | **Reverse primer** | **Sequence (5’-3’)** |
| --- | --- | --- | --- | --- | --- |
| ***Phytophthora infestans*** | *ActA* | PiActin-F | CATCAAGGAGAAGCTGACGTACA | PiActin-R | GACGACTCGGCGGCAG |
|  | *HMP1* | PiHMP1-F | CATGATGGCTGTCATGGTCGGTGAGG | PiHMP1-R | TTAGCTAACATTCAAGCGAGCATGAAG |
|  | *NPP1* | PiNPP1-F | CAGATCCACATCTCGAACGGCT | PiNPP1-R | CTGTAGCCGTCGATCGTGTTG |
|  | *CDC1*4 | PiCDC14-F | GGAAGCGATCGAGTTCCTCCC | PiCDC14-R | TTCTCGACCCGTTCATAGTG |
|  | *IPI-B* | IPI-B-F | GTAATGCTGCTGTCGGCGCTG | IPI-B-R | GCCGTTCTGCGACGTAGATGTG |
|  | *IPI-O* | IPI-O-F | CTCGCACTCAGTCCAAGACGG | IPI-O-R | AGCCTTATCGAGATGCTTGGTCC |
|  | *Avr1* | Avr1-F | CCGTCGTTTATCGAGTCCTTCG | Avr1-R | CGCCAGGCTCGTTCCG |
|  | *Avr2* | Avr2-F | ATGCGTCTCGCCTACATTTT | Avr2-R | GCATTTGAAAATTAAGGGTGACA |
|  | *Avr3* | Avr3a-F | CGCCATAAACTTTGCAACCA | Avr3a-R | TGCCGGCTGAATCGTGTAT |
|  | *Avr4* | Avr4-F | CATCACGACGCTTAAACTCAACGG | Avr4-R | GATACAACTTGACATACTTCAGGAGCG |
|  | *Avrblb2* | Avrblb2-F | CGTCGCAGCATTCCCAAT | Avrblb2-R | GCCACAGTGTCAGGAGATGTCTT |
|  | *PITG_03192* | Pi03192-F | CCTGCTCGTCGTCAGTGTGA | Pi03192-R | TCGAAATTCCGGTTATTCATGA |
| ***P. capsici*** | *HMP1* | PcHMP1-F | CATGATGGCAGTCATGGTCGGTGAAG | PcHMP1-R | TTAGCTAACATTGAGGCGGGCATGCAG |
|  | *NPP1* | PcNPP1-F | CAGCTCCACATCACCAACGGCT | PcNPP1-R | CTCTTCCCGTTCAAATAGTTC |
|  | *CDC1*4 | PcCDC14-F | GGAAGCGATTGAGTTCTTGC | PcCDC14-R | TTCTCCACACGCTCAAAGTG |
| ***P. sojae*** | *HMP1* | PsHMP1-F | CATGATGGCCGTCATGGTCGGCGAG | PsHMP1-R | TTAGCTAACATTCAGACGAGCGTGCAG |
| ***Solanum lycopersicum*** | *ActA* | Actin-F | TATGGAAACATTGTGCTCAGTGG | Actin-R | CCAGATTCGTCATACTCTGCC |
|  | *Chi3* | Chi3 -F | CAATTCGTTTCCAGGTTTTG | Chi3-R | ACTTTCCGCTGCAGTATTTG |
|  | *Chi9* | Chi9-F | AATTGTCAGAGCCAGTGTCC | Chi9-R | TCCAAAAGACCTCTGATTGC |
|  | *PR1a* | PR1A-F | TCTTGTGAGGCCCAAAATTC | PR1A-R | ATAGTCTGGCCTCTCGGACA |
|  | *PR1b* | PR1A-R | ATAGTCTGGCCTCTCGGACA | PR1B-R | GAACCTAAGCCACGATACCA |
|  | *PR2a (GlucA)* | PR2A-F | TATAGCCGTTGGAAACGAAG | PR2A-R | TGATACTTTGGCCTCTGGTC |
|  | *PR2b (GlucB)* | PR2B-F | CAACTTGCCATCACATTCTG | PR2B-R | CCAAAATGCTTCTCAAGCTC |
|  | *PR5* | PR5-F | ATGGGGTAAACCACCAAACA | PR5-R | GTTAGTTGGGCCGAAAGACA |
|  | *HSR203J* | HSR203J-F | TCCCGTCATTCTTCACTTCC | HSR203J-R | GTTGAAATCGGCGTATTCGT |
|  | *P69a/b* | P69a/b-F | GTTTGCAGCAGAACATGGGTGTATGGAA | P69a/b-R | CTCCTGCAGCTGTGCTTGCTGTGTGTGTAC |
|  | *P69c* | P69c-F | GGAATCTCAGCTGAGAGGTC | P69c-R | GAAGCCATGAAAGAACATTGTC |

^a^:F: forward primer, R: reverse primer
